# Comparison of specialist ataxia centres with non-specialist services in terms of care access and organisation, health services resource utilisation and costs in Germany using patient-reported data

**DOI:** 10.1016/j.heliyon.2025.e42121

**Published:** 2025-01-29

**Authors:** Julie Vallortigara, Julie Greenfield, Barry Hunt, Holm Graessner, Carola Reinhard, Andreas Nadke, Bart-Jan Schuman, Deborah Hoffman, Steve Morris, Paola Giunti

**Affiliations:** a Ataxia Centre, Department of Clinical and Movement Neurosciences, UCL Queen Square Institute of Neurology, Queen Square House, Queen Square, London, WC1N 3BG, UK; b Ataxia UK, London, UK; c Centre for Rare Diseases and Institute of Medical Genetics and Applied Genomics, University Hospital Tübingen, Tübingen, Germany; d Deutsche Heredo Ataxie Gesellschaft (DHAG), Hofener Straße 76, 70372, Stuttgart, Germany; e Friedreich Ataxie Förderverein e.V., Am Heckenacker 9a, 85652, Pliening, Germany; f Takeda Pharmaceuticals, Cambridge, MA, USA; g Primary Care Unit, Department of Public Health and Primary Care, University of Cambridge, Cambridge, UK

**Keywords:** Ataxia, Specialist centre, Care pathway, Health service use, Costs

## Abstract

**Background:**

The ataxias are rare complex neurological disorders challenging to diagnose and manage. We explored the patient pathways, health care use and costs of individuals attending a specialist ataxia centre (SAC) compared with non–specialist settings in Germany.

**Methods:**

We distributed a survey to people with ataxia to gather information about diagnosis and management of the ataxias, utilisation of health care services, and patients’ satisfaction in both SAC and non-specialist settings. We compared mean resource use and health service costs per patient, stratifying by whether patients attended a SAC or not.

**Results:**

We collected and analysed responses from 101 participants. For patients who visited both a SAC and a standard neurology clinic, 67.2 % reported that the care received at a SAC was better. Positive feedback about SAC services included understanding their condition (66.7 % positive feedback), giving practical advice to manage better their ataxia (66.7 % positive feedback) and offering opportunities to take part in research (79.2 % positive feedback). Costs were not significantly different between those attending a SAC and those who did not, although the average cost per patient per year found in SACs was higher compared with non-SACs. The mean total cost per patient over a one-year period was €2091 for non-SAC patients and €4043 for SAC patients (P = 0.19 in adjusted analyses). We identified barriers in accessing SACs including the lack of clear referral pathway and the travel required to such centres. We made recommendations about gaps identified in the care provided based on people's experience and feedback.

**Conclusions:**

This study provides useful information about ataxia patient care pathways in Germany, with higher patients’ satisfaction in SAC compared to non-SAC, without a significant difference in costs. The findings can be used to change policy for better care for people with these rare complex neurological diseases.

## Highlights

• A patient-centred study on people's experience and feedback on treatment and care.
• Survey on diagnosis and management of the ataxias in secondary care settings.
• Increase the care for ataxia patients with a clear referral pathway and telemedicine.
• Ensure provision and funding for multidisciplinary clinic within the SAC service.
• Coordinate the care outside of SAC with a holistic approach to manage better at home.

## 1 Introduction

The ataxias are a group of complex rare neurological disorders affecting motor functions. Patients with ataxia experience lack of balance and coordination, as well as slurred speech and impaired eye movements among other symptoms [1]. Patients often become wheelchair-bound, and the level of disability can progress leading to dependency [2]. Communication becomes impaired due to speech disturbances. Other symptoms are associated with specific ataxia conditions, these include spasticity, tremor, sensory disturbance, auditory and visual impairment, bladder and bowel dysfunction, cardiac complications, musculoskeletal complications, and cognitive impairment.

The ataxias are clinically and genetically heterogenous. The conditions covered in this study are disorders where ataxia is the primary symptom due to progressive inherited and idiopathic ataxias. Epidemiological studies have estimated an overall ataxia prevalence rate of 26 cases per 100,000 in children, and 2.7 to 3.3 cases per 100,000 for hereditary cerebellar ataxia (CA) in adults [3]. At least one hundred genetic types of ataxias have been identified [4,5]. Some of the progressive ataxias are rare and therefore not well known. However, Friedreich's ataxia (FA), the most common inherited ataxia to date, have been well described. The estimated incidence rate of FA is 1 in 29,000 amongst Caucasians [6] with a prevalence of 1 in 47,000 in Germany [7]. Epidemiological data for each European country is still lacking.

Rare neurological disorders like ataxias are challenging to manage due to a lack of awareness and understanding among healthcare professionals (HCPs), hence the input of specialists is crucial for diagnosis and treatment [8,9]. Being able to reach diagnosis in a timely manner and provide an early intervention for ataxia could reduce complications and disabilities. To date, there are no effective treatments for most forms of ataxia but there have been advances in interventions including physiotherapy and occupational therapy [10,11]. Recently, Omaveloxolone, a Nrf 2 activator, has been approved by the FDA and the EMA for the treatment of FA [[12], [13], [14]]. Ataxia management requires a broad and multidisciplinary approach [10], with a complex care delivered to patients by a multidisciplinary team (MDT), including neurologists, geneticists, physiotherapists, occupational therapists, speech and language therapists, and neuropsychologists [15]. Specialist ataxia centres (SACs) should be able to provide coordinated care including diagnosis services and address ataxia patients’ specific needs. In Germany, there is currently no formal national recognition or qualification process for being a SAC, as opposed to the UK where the charity for ataxia patients, Ataxia UK, has taken the lead in awarding the title of Centre of Excellence based on a strict set of criteria. Therefore, in Germany, being a SAC means an ataxia outpatient clinic at a University Hospital focusing on research with access to ataxia healthcare specialists, innovative diagnostic (in particular genetic) methodologies and ad hoc multidisciplinary expertise. According to the German Reference Network for Rare Neurological Diseases, there are nine SACs across the country [16].

This project explores the patient pathways of individuals with progressive ataxias and the health economic effects of SACs compared with non-specialist settings in Germany. This study was part of a larger project including three European countries, UK, Germany and Italy; some of the results of which were recently published [17,18]. We collected information using a patient survey and analysed patients’ experience and satisfaction in attending healthcare services in the context of the diagnosis and management of their ataxias. Analysis of the cost-effectiveness was carried out by comparing the use of ataxia specialist centres with non-specialist services. We also investigated the role of SAC service in terms of outcomes for patients, and the impact of comorbidities and complexity of their ataxia on the resource use and costs. The goal of this study is to inform policy recommendations on how to improve treatment and care for people with these very rare and complex neurological diseases.

## 2 Material and methods

We designed and conducted a survey to gather patients' experiences of diagnosis and management of the ataxias whether they were patient in a SAC or not. For the preparation of the survey in Germany a focus group was created including German ataxia patient organisations’ DHAG and Ataxie Forderverein e.V. representatives and members of the German centres of the European Reference Network for Rare Neurological Diseases (ERN-RND). This focus group identified nine German SACs located at the University Hospitals in: Luebeck, Munich, Tuebingen, Bonn, Essen, Aachen, Berlin, Düsseldorf, Magdeburg.

### 2.1 Patients

The population of interest for participation in the survey was all patients (or carers of patients, as proxy) with ataxia who were 16 years old or over, as the SACs are for adults, and lived in Germany. This age group reflects the ages of patients seen at the adult SACs. As the survey was distributed by patient groups in Germany, (DHAG and Ataxie Forderverein e.V.), with the support of the clinicians working at the SACs, the target population was individuals who are members of such groups (comprising people with progressive ataxias such as FA, inherited CA, sporadic or idiopathic CA).

### 2.2 Survey design

The survey was initially designed by a working group comprised of Costello Medical Consulting staff, Ataxia UK patient representatives, a specialist ataxia neurologist, a specialist ataxia nurse, a health economist and representatives of the pharmaceutical industry. After review with the focus group members, the final survey was constructed of 54 non-obligatory questions relating to the following topics: demographics, diagnosis, referrals, patient encounters with HCPs, ataxia symptoms experienced, treatments received and patient satisfaction. The context of the study and the medical terms used were explained at the beginning of the survey (see survey form in Appendix 1).

### 2.3 Data collection

A weblink was used to direct participants to SurveyMonkey – an online platform which hosted the survey. The size of the targeted population is unknown as patient group members as well as non-members could have taken part in the study. The survey was rolled out between February and October 2020. Survey responses were exported from SurveyMonkey and collated in Microsoft Excel, where the raw data were cleaned and analysed. The data cleaning process involved the removal of all respondents who did not answer positively to all three screening questions (questions 3,4,5; see Appendix 1). Where the respondent gave clear contradictory responses, the responses to those questions were removed from the analysis. Incomplete surveys were not removed from the analysis, as respondents chose to answer some questions and to skip others. The number of respondents was reported for each individual question.

### 2.4 Ethics and consent

The survey was anonymised therefore no ethical approval was needed to roll out the survey in Germany. Consent was obtained online, with the act of submitting the survey considered as consent to take part in the study.

### 2.5 Data analyses

Responses to survey questions were stratified by the following characteristics and the distribution of eligible responses determined for each stratification independently: attendance at a SAC, comorbidities, and complexity of the disease. For the attendance at a SAC (questions 25 and 35; Appendix 1) people who had never been to a SAC were grouped in the ‘non-SAC’ group; people who are currently going to a SAC were grouped in ‘SAC’ group. There was a third group of people who used to go to a SAC but no longer attend; they were grouped in the ‘USED to SAC’ group. This group was not included in the statistical analysis because they experienced a mixed care pathway. However, the results of the survey for this group have been used to identify barriers to continuing to access SACs. For comorbidities, answers to question 6 (Appendix 1) were used for stratification. People who answered “none” were put in the group with nocomorbidity on top of their ataxia. People who reported other conditions were put in the group with one or multiple comorbidities due to living with at least one other condition on top of their ataxia. For complexity of the disease, answers to question 43 (Appendix 1) were used to create three groups. People who did not report any of the symptoms listed in that question were put in group 0. People who reported between one and four symptoms associated with their ataxia were put in group 1 (low level of complexity). People who reported between five and eight symptoms were put in group 2 (moderate level of complexity).

In questions with responses measured on a Likert scale, the responses were grouped into two categories, where affirmative responses were considered to be ‘very positive/positive/slightly positive’ or ‘very effective/effective/slightly effective’ or ‘strongly agree/agree/slightly agree’ and negative responses were considered to be ‘very negative/negative/slightly negative’ or ‘very ineffective/ineffective/slightly ineffective’ or ‘strongly disagree/disagree/slightly disagree’. For questions 51–52, affirmative responses were considered to be ‘best it could be/very well/quite well/adequately’. Results were prepared as tabulated descriptive statistics and presented as numbers (n) and percentage (%) of total respondents per question.

The questions were treated independently, and the Chi-Square Pearson test was performed to compare results between the ‘SAC’ and ‘non-SAC’ groups for each question.

### 2.6 Resource use and costs

Participants were asked to record the number of health care contacts they had received in the preceding 12 months, specifically due to their ataxia. Participants were asked to record the number of contacts with the family doctor, visits to see a resident neurologist/outpatient visits to a neurologist in a hospital with a general neurological department, SAC visits, hospital inpatient stays, emergency room visits, physiotherapy appointments, speech and language therapy appointments, occupational therapy appointments, and other consultant specialist visits (e.g. an ophthalmologist, an ear, nose and throat specialist, urologist, gastroenterologist and others). Unit costs for each type of contact were obtained from local providers or published sources [19]. All costs were calculated in 2022 € using GDP Purchasing Power Parities.

We compared average resource use for each contact type, and costs, over a 12- month period, stratifying patients by whether they were currently attending a SAC or had never attended a SAC. We then repeated this analysis, additionally stratifying by the presence of comorbidities (no/yes) and number of symptoms experienced as a result of ataxia (0, 1–4, 5–8 symptoms). We tested for significant differences in mean resource use and costs between the SAC/non-SAC groups using unadjusted and adjusted ordinary least squares regression analysis, the latter controlling for age, sex, plus the number of symptoms experienced as a result of ataxia and whether or not the patient had comorbidities, as appropriate. We also tested for significant differences in mean contacts and costs between sub-groups of patients by the presence of comorbidities and number of symptoms experienced separately for non-SAC and SAC groups using adjusted ordinary least squares regression analysis.

As part of the survey, we also asked respondents who reported using the SAC to record the main mode of transport they used to travel to the centre, as well as the travel time from home to the centre. We also asked all respondents how long it took to travel one-way to receive ataxia care by a neurologist based in a general neurology clinic (not at a SAC).

All analyses for resource use and costs were performed using Stata software [20].

## 3 Results

### 3.1 Cohort demographics

We had 101 respondents to the survey and the cohort demographics are summarised in Table 1. Participants reported a range of diagnoses including inherited CA (55.3 %), other types (16 %), FA (14.9 %), and unknown types (13.8 %). People's answers showed a wide spread of mobility with ataxia, from no impairment to being bedridden. The top three responses were moderate impairment with walking aids (21 %), mild impairment (20 %), and using walking aids indoors and wheelchair outdoors (19 %) (Supplementary Table 1). More than half of the respondents (53.6 %) said that their ataxia causes permanent problems and restricts their activities most of the time, followed by 25.8 % experiencing frequent problems and limitations in their activities (Supplementary Table 2). The burden of ataxia on people's lives progressed with time, over the years and decades since their diagnosis (Supplementary Fig. 1 and Table 3). Looking at the attendance to a SAC, 48 participants are currently going (57.1 %), 13 used to go to a SAC (15.5 %), and 23 have never been (23.4 %) (Table 1).

**Table 1:** Survey respondent demographics.

Respondent type (n = 101)	patient	90 (89.1 %)
	On behalf of the patient	11 (10.9 %)
Age distribution (n = 101)	16–29	12 (11.9 %)
	30–59	60 (59.4 %)
	60–80	29 (28.7 %)
	80+	0 (0 %)
Gender (n = 101)	Female	48 (47.5 %)
	Male	53 (52.5 %)
Diagnosis (n = 94)	FA	14 (14.9 %)
	Inherited CA	52 (55.3 %)
	Other types	15 (16 %)
	Not known	13 (13.8 %)
Attendance to SAC (n = 84)	Non-SAC	23 (23.4 %)
	SAC	48 (57.1 %)
	Used to SAC	13 (15.5 %)

CA, cerebellar ataxia; FA, Friedreich's ataxia. Other types: no progressive ataxia, episodic ataxia, multiple system atrophy cerebellar type C, cerebellar oculomotor disorder, ataxia with oculomotor apraxia type 2, sporadic ataxia of unknown aetiology, autoimmune response, leukodystrophy, cerebellar ataxia and mitochondriopathy, orthostatic tremor.

### 3.2 Getting a diagnosis

We asked participants if they had a confirmed diagnosis which involved genetic or other tests: 78.3 % of our cohort have a confirmed diagnosis versus 16.3 % who don't (Supplementary Table 4). These diagnoses were given in a SAC for 41.2 %, in a non-SAC neurology clinic for 29.4 %, and in other healthcare services for 27.1 % (Supplementary Table 5). There is a greater diversity of confirmed diagnoses in the SAC group compared to the non-SAC group (Supplementary Fig. 2). Looking at the time it took for people to obtain a specific diagnosis after seeing a neurologist, we note that SACs do not perform better (Supplementary Figure 2bis).

### 3.3 Patient pathway

#### 3.3.1 Referral to see a neurologist

When we asked participants how long they waited to see a neurologist after they sought medical advice for their ataxia symptoms, almost half saw a neurologist within 6 months (48.3 %) (Supplementary Table 6). For the place where participants went for this referral, 68.2 % of participants went to a non-specialist clinic as a first referral, versus 17.7 % who went to a SAC as a first referral (Supplementary Table 7).

#### 3.3.2 Access to a SAC

Participants reported different ways of accessing a SAC: 53.9 % were referred by a neurologist from a non-specialist neurology clinic, 26.2 % by the family doctor, and 13.9 % reported alternative ways to get access to a SAC (see caption of Table 2). These include accessing a SAC by being engaged in research studies and by making a self-referral. Around half of the people in the ‘SAC’ and ‘Used to SAC’ groups went to a non-specialist neurology clinic before going to a SAC (Supplementary Table 8). For those who never attended a SAC, the top reasons were that the local care received is sufficient (18.2 %) or that the referral was not offered (18.2 %), followed by other reasons (see caption Table 3), including the lack of a clear pathway for referral and the focus of SAC on research and clinical studies (14.5 %). Another barrier to accessing a SAC reported was the distance to travel to a centre. This was one main reason why people stopped going to a SAC (16.7 %) (Supplementary Table 9). On average, participants who visited a SAC travelled for longer than those who visited a standard neurology clinic: 22.6 % travelled less than 1 h to reach a SAC, versus 67.4 % under 1 h for non-SAC visits (Supplementary Figs. 3 and 4). Among the other reasons why people stopped going to a SAC, participants also highlighted that the local care received was equivalent (13.3 %), and for other reasons (33.3 %), which again included the mention of focus of SAC on research studies (Supplementary Table 9).

**Table 2:** Who referred the participants to a SAC.

Answer choices	Responses N (%)
GP	17 (26.15 %)
Neurologist	35 (53.85 %)
Other	9 (13.85 %)
Unsure	4 (6.15 %)
**Total**	**65 (100** **%)**

Other ways: self-arrangement due to EFACTS (European Friedreich's Ataxia Consortium for Translational Studies), self-referral with ataxia in the family, personal connections, neurologist with own practice, mother with ataxia and took part in the study for non-examined relatives, GP referred in according to recommendation by Prof Schoels, social-paediatrist centre, was interviewed by SCA3 runs in the family, no referral as arranged appointment myself, heard of the clinic from relatives and arranged an appointment myself.

**Table 3:** Reasons why people never visited a SAC.

Reasons	Responses N (%)
Current level of care is sufficient	10 (18.2 %)
I asked to be referred to a SAC but was refused by my doctor	0 (0 %)
SACs are too far away for me to travel to	3 (5.4 %)
Did not wish to be referred	0 (0 %)
A referral to SAC was not offered to me	10 (18.2 %)
Other [please specify in the text box]	8 (14.5 %)
Not applicable	20 (36.4 %)
Unsure	4 (7.3 %)
Do not wish to answer	–
Total N respondents	55 (100 %)

#### 3.3.3 Access to an MDT

Only 16.5 % participants replied that they did see an MDT for their ataxia (Supplementary Table 10A). The majority of the referrals to an MDT clinic were done by a neurologist at the local hospital (45.5 %) or by a family doctor (family doctor being most of the other referral types in the ‘Other’ response) (Supplementary Table 10B). Among the people who went to an MDT, 68.8 % found the visit to that clinic to be effective (Supplementary Table 10C).

#### 3.3.4 Patient experience and satisfaction

When we asked participants whether the care received at a SAC was an improvement compared to the care received in a standard neurology clinic, 67.2 % reported that the care received at a SAC was better (Supplementary Table 11). More specifically, when comparing the SAC and non-SAC services, people reported that SACs do better in understanding condition (66.7 % positive feedback), in helping people to cope with their condition (60.8 % positive feedback), in giving practical advice on how to live better with ataxia (66.7 % positive feedback), in providing medical advice on the management of the ataxia symptoms (58 % positive feedback), and in offering opportunities to take part in research (79.2 % positive feedback). The SACs were not better in coordinating referrals to other healthcare professionals (50 % neutral feedback) and in communications with social care professionals (55.8 % neutral feedback) (Table 4).

**Table 4:** Participants feedback on the comparison of the SAC and non-SAC services.

Germany Group N (%)	YES to SAC			Used to SAC			Total		
Feedback	Positive	Neutral	Negative	positive	Neutral	Negative	Positive	Neutral	Negative
Good understanding of your condition									
	31 (75.6)	10 (24.4)	0 (0)	3 (30)	6 (60)	1 (10)	34 (66.7)	16 (31.3)	1 (2)
Total	41 (100)			10 (100)			51 (100)		
Makes you able to cope better with your condition									
	28 (68.3)	13 (31.7)	0 (0)	3 (30)	6 (60)	1 (10)	31 (60.8)	19 (37.2)	1 (2)
Total	41 (100)			10 (100)			51 (100)		
Gives practical advice about how to live better with ataxia									
	30 (75)	10 (25)	0 (0)	4 (36.4)	6 (54.5)	1 (9.1)	34 (66.7)	16 (31.3)	1 (2)
Total	40 (100)			11 (100)			51 (100)		
Provides medical advice on the management of your symptoms									
	27 (67.5)	13 (32.5)	0 (0)	2 (20)	7 (70)	1 (10)	29 (58)	20 (40)	1 (2)
total	40 (100)			10 (100)			50 (100)		
Coordination of referrals to specialists									
	19 (50)	17 (44.7)	2 (5.3)	1 (12.5)	6 (75)	1 (12.5)	20 (43.5)	23 (50)	3 (6.5)
Total	38 (100)			8 (100)			46 (100)		
Offers to participate in research									
	34 (85)	5 (12.5)	1 (2.5)	4 (50)	3 (37.5)	1 (2.5)	38 (79.15)	8 (16.65)	2 (4.15)
Total	40 (100)			8 (100)			48 (100)		
Liaising with social workers									
	16 (44.4)	20 (55.6)	0 (0)	2 (28.6)	4 (57.1)	1 (14.3)	18 (41.9)	24 (55.8)	1 (2.3)
Total	36 (100)			7 (100)			43 (100)		

Participants were asked if the HCPs they visited in primary care understood how to manage their condition: 27 responded positively (43.5 %), 25 were neutral (40.3 %) and 10 responded negatively (16.2 %). When asked if the HCPs knew about the treatments available for their ataxia, 28 responded positively (43 %), 24 were neutral (37 %) and 13 responded negatively (20 %) (Supplementary Tables 12a–b). When asked the same about secondary care visits, 46 responded positively (76.7 %), 11 were neutral (18.3 %) and 3 responded negatively (5 %) regarding whether HCPs understood how to manage their condition. Then, 45 responded positively (77.6), 11 were neutral (19 %) and 2 responded negatively (3.4 %) regarding whether HCPs knew about the treatments available for their ataxia (Supplementary Tables 12c–d). When the same question was asked about SAC services, 45 responded positively (95.7 %), 2 were neutral (4.3 %), and 0 responded negatively (0 %), regarding whether HCPs understood how to manage their condition. Finally, 45 responded positively (95.7 %), 2 were neutral (4.3 %), and 0 responded negatively (0 %) regarding whether HCPs knew about the treatments available for their ataxia (Supplementary Tables 12e–f).

### 3.4 Gaps-unmet needs

We asked people what was needed in order to improve the care they received for their ataxia. The results are summarised in Table 5. People want to see improvement in the information shared about their condition and potential treatment, they wish to feel more in control, receive better advice on how to cope, and gain better access to holistic treatments. Managing the condition at home with more help around adaptations, and ways to receive personalised care locally, was also valued by the participants. When we looked at the difference in feedback between people who went to a SAC and people who did not, those in the SAC group have more needs for improvements compared with those in the non-SAC group (Supplementary Table 13). Also, people in the SAC group have more needs specifically about communications to the employer, care at home, home adaptation, and advice on living with ataxia.

**Table 5:** Participants feedback on how to improve the care delivered.

Answer choices	Responses N	Responses (%)
More information about my condition	25	38.46
More help so I can feel in control of my disease (to cope better)	27	41.54
Knowing my specific diagnosis earlier	14	21.54
Better management of my symptoms	22	33.85
Better practical advice on living with my condition	29	44.62
Better access to therapies (physiotherapy, speech therapy, occupational therapy)	20	30.77
More information on help adapting my home	20	30.77
Help in communicating with my employer	7	10.77
More information about the genetics of my condition/whether my children or grandchildren are at risk of inheriting ataxia	10	15.38
Continuing the same level of care in my home if I am not longer able to visit an ataxia specialist centre	15	23.08
I am satisfied with my care and do not need improvement	9	13.85
Other please specify	7	10.77
I do not know	1	1.54

### 3.5 Resource use and costs

The most common contacts for SAC patients and non-SAC patients were visits to physiotherapy (mean 27.9 and 33.7 visits per patient per year respectively), speech and language therapy (11.5 and 8.9 respectively) and occupational health therapy (10.4 and 7.9 respectively) (Table 6). Other than the SAC visits themselves, the differences in the numbers of contacts for the different types of health service use between the SAC and non-SAC groups were mostly non-significant in both unadjusted and adjusted analyses. One exception was speech and language therapy visits, with a higher mean contact for patients in the SAC group compared to the non-SAC group in the adjusted analysis (P = 0.02). The mean total cost per patient over a one-year period was €2091 for non-SAC patients and €4043 for SAC patients (P = 0.19 in adjusted analyses). We note that a visit to see a neurologist in a SAC in Germany is substantially more expensive than seeing a neurologist in a standard neurology clinic. Together with the costs of inpatient stays and emergency room visits, these could explain the difference (non-significant) in mean cost per patient per year between SACs and non-SACs.

**Table 6:** Health care contacts over a one-year period for non-SAC and SAC patients.

**Health care contacts**	Patients who reported never attending a SAC				Patients who reported attending a SAC currently				**Unit cost**	**P-value**^a^	**P-value**^b^
	**N**	**Mean**	**Std. Dev.**	**Median**	**N**	**Mean**	**Std. Dev.**	**Median**			
Specialist centre visits	10	0.0	0.0	0	33	1.3	0.9	1	170	<0.01	0.01
Family doctor visits	8	5.5	10.1	2	34	4.9	5.2	3.5	29	0.80	0.23
Neurologist visits	11	3.7	3.1	3	30	2.5	2.9	2	65	0.23	0.47
Inpatient stays	10	0.0	0.0	0	31	0.3	0.7	0	4950	0.24	0.24
Emergency room visits	9	0.1	0.3	0	31	0.6	2.7	0	144	0.59	0.92
Physiotherapy visits	11	33.7	22.8	50	28	27.9	23.7	40	38	0.49	0.92
Speech and language therapy visits	10	8.9	18.5	0	33	11.5	17.2	1	38	0.69	0.02
Occupational health therapy visits	10	7.9	17.2	0	30	10.4	17.7	0	38	0.70	0.41
Other consultant specialist visits	9	1.6	2.3	1	28	1.6	2.0	1	65	0.95	0.54
Total cost	6	2091	1449	2291	23	4043	3997	2830		0.26	0.19

SAC, specialist ataxia centre; N, number of participants who responded to that question.

a Test for significant differences in mean values between Non-SAC and SAC groups (unadjusted).

b Test for significant differences in mean values between Non-SAC and SAC groups (adjusted for age, sex, number of symptoms and number of comorbidities).

When we stratified our analyses by the presence of comorbidities, these trends remained the same, except we also found evidence of significantly lower neurologist (in a non-SAC setting) and family doctor visits in the SAC group compared to the non-SAC group among people with no comorbidities (P = 0.04 and P = 0.03 respectively; Table 7). The mean total cost per patient over a one-year period was €2998 for non-SAC patients with no comorbidities and €3321 for SAC patients with no comorbidities (P = 0.63), and €1638 for non-SAC patients with comorbidities and €4788 for SAC patients with comorbidities (P = 0.40). Similarly, the difference in costings between the non-SAC and SAC groups with comorbidities can be explained by the costs of inpatient stays. Among patients with comorbidities who did not attend a SAC there were no differences in the mean number of contacts for any types of resource use, or for costs; the same was true for patients in the SAC group.

**Table 7:** Health care contacts over a one-year period for non-SAC and SAC patients by presence of comorbidities.

**Health care contacts**	Patients who reported never attending a SAC				Patients who reported attending a SAC currently				**P-value**^a^	**P-value**^b^
	**N**	**Mean**	**Std. Dev.**	**Median**	**N**	**Mean**	**Std. Dev.**	**Median**		
**No comorbidities**										
Specialist centre visits	3	0.0	0.0	0	10	1.7	1.6	1	0.10	0.09
Family doctor visits	4	9.0	14.2	3	11	4.5	4.5	3	0.34	0.04
Neurologist visits	4	5.8	4.3	5	10	1.9	1.3	2	0.02	0.03
Inpatient stays	3	0.0	0.0	0	10	0.1	0.3	0	0.61	0.33
Emergency room visits	3	0.0	0.0	0	10	0.0	0.0	0	–	–
Physiotherapy visits	3	33.3	28.9	50	10	34.0	23.7	50	0.97	0.33
Speech and language therapy visits	3	17.3	28.3	2	10	13.9	18.4	5	0.80	0.25
Occupational health therapy visits	3	0.0	0.0	0	10	9.9	17.1	0	0.35	0.78
Other consultant specialist visits	2	0.0	0.0	0	10	1.2	0.9	1	0.11	0.16
Total cost	2	2998	888	2998	10	3321	1316	2924	0.75	0.63
**One or more comorbidity**										
Specialist centre visits	6	0.0	0.0	0	19	1.1	0.3	1	0.00	0.00
Family doctor visits	4	2.0	1.8	2	18	4.4	5.7	3	0.42	0.61
Neurologist visits	6	2.2	1.2	2	17	2.5	3.3	2	0.83	0.31
Inpatient stays	7	0.0	0.0	0	18	0.3	0.8	0	0.31	0.41
Emergency room visits	6	0.2	0.4	0	18	1.1	3.5	0	0.55	0.91
Physiotherapy visits	7	32.3	23.7	50	15	27.5	24.2	40	0.67	0.70
Speech and language therapy visits	6	0.0	0.0	0	19	10.3	17.1	1	0.16	0.15
Occupational health therapy visits	6	5.0	12.2	0	17	9.6	17.0	0	0.55	0.37
Other consultant specialist visits	7	2.0	2.4	1	14	1.9	2.7	1	0.91	0.30
Total cost	4	1638	1554	1416	10	4788	5648	2473	0.30	0.40
**P-value**^c^										
Family doctor visits	0.019				0.933					
Neurologist visits	0.017				0.830					
Inpatient stays	–				0.981					
Emergency room visits	–				0.344					
Physiotherapy visits	0.538				0.440					
Speech and language therapy visits	–				0.234					
Occupational health therapy visits	0.500				0.451					
Other consultant specialist visits	0.379				0.564					
Total cost	0.656				0.995					

A hyphen “-” indicates that the parameter is not estimable, due to small numbers of observations.

SAC, specialist ataxia centre; N, number of participants who responded to that question.

a Test for significant differences in mean values between Non-SAC and SAC groups (unadjusted).

b Test for significant differences in mean values between Non-SAC and SAC groups (adjusted for age, sex and number of symptoms).

c Test for significant differences in mean values by comorbidities separately for Non-SAC and SAC groups (adjusted for age, sex and number of symptoms).

We also examined differences in costs by the number of symptoms related to ataxia. The numbers in the stratified groups were small so caution is needed when examining these results. For further details please see supplementary data (Supplementary Table 14).

Among those who attended a SAC, the modal travel time was 1–2 h (Supplementary Table 15), though a small proportion of patients travelled for more than 3 h. The most common mode of transport was by car. For the vast majority of patients who did not attend the SAC (86 %) the travel time to see the neurologist at the general neurology clinic was less than 1 h. In SAC group, 63 % of patients reported that the travel time to see the neurologist at the general neurology clinic was less than 1 h. If ‘unsure’ and ‘not applicable’ responses were removed this increased to 75 % (30/40 respondents).

## 4 Discussion

The aim of this study was to investigate the care pathways of patients living with ataxia in Germany, and to better understand the role of SACs in the management of the ataxias compared to standard neurology clinics. We also wanted to identify unmet needs in the treatment and care delivered to people with ataxia in Germany.

### 4.1 Patient care pathways and satisfaction

The results revealed there was no systematic referrals to SAC in Germany. A neurologist was seen by 48.3 % of participants within 6 months after first medical enquiry for ataxia symptom. This was similar to the survey results in the UK [21] and in Italy (Vallortigara J et al., in preparation). However, only 18 % went to a SAC directly for that referral. The lack of formal referrals pathways in Germany like in Italy and the UK reflects (i) a non-recognition by the healthcare system of Specialist Centres in terms of care and referral pathways and funding as well as (ii) limitation due to the regional organisation of the healthcare system. Interestingly, based on the feedback from participants, attendance at a SAC did not result in a shorter time to reach a diagnosis. This might be due to the large spectrum of diagnoses including more complex cases being seen at a SAC, and only more common ataxia types being seen in non-SAC setting. However, the patient experience and satisfaction of SAC visits was better when compared to primary and secondary care services in terms of level of knowledge and expertise on the ataxias. This is not surprising as the ataxia healthcare providers are actively participating in research and therefore can translate timely new knowledge into the management of the ataxias. Overall, 67 % of those who accessed a SAC and a non-SAC service reported that the care received in the SAC was better. More specifically, the SAC service made a difference to these people in terms of understanding the condition, giving practical advice on how to live better with ataxia and offering opportunities to take part in research. Barriers in accessing and retaining patients at a SAC - including the lack of systematic referral process and the travel required - are a concern. Moreover, the fact that 68 % of those who attended a SAC travelled by car, with 72 % travelling for 1–3 h, could play a role on the level of fatigue patients experience with travelling to access a SAC.

Only a minority of participants went to an MDT (12 %) for the management of their ataxia as this is not offered as a standard care service. Among them 69 % of these people were satisfied with their visit to an MDT. This is a lower proportion of people accessing MDT clinics compared to the UK where a third of the cohort went to an MDT [21]. In that study, the effectiveness of visits to an MDT was also reported by participants.

### 4.2 Role of SACs and MDT clinics

The SAC visit is an outpatient neurology clinic at a University Hospital where patients go to once a year or even less often (personal communication with patients’ groups DHAG and Friedreich Ataxie Förderverein eV representatives and ERN-RND coordinators in Germany). This defines a role and use of the SACs in Germany that are distinct from the UK system [21], in Germany the SAC focus on research and not on the management of the symptoms and care coordination. Ideally the ataxias should be treated by a highly specialised MDT [22,23]. The care model of SAC with MDT clinics was created first in the UK and has now been adopted in the USA [8,9,24]. SACs in Germany are not based on that model. Indeed, SAC facilitate access to MDT clinics and therefore are not being reimbursed in the SAC outpatient setting.

### 4.3 Costs analysis

We showed in a previous study on FA in Germany that the burden of the condition is significant on the person's quality of life, together with the caregivers, health cost and the impact on society (due to work loss) [25]. Our cost analysis in this study found a trend towards highest costs per patient per year found in SACs compared with non-SACs. This was also the case for patients with comorbidities, with a trend towards higher cost per patient per year for SAC patients compared with non-SAC patients. In terms of number of healthcare visits, the only significant difference was that SAC patients had more visits to speech and language therapists. This might reflect better management of the speech symptoms. Interestingly, patients with no comorbidities who were in the SAC group went less frequently to the family doctor or the neurologist within a non-SAC setting. This is possibly due to the more comprehensive approach in SAC compared to non-SAC reducing the need of additional HCPs interventions. The combination of the results of the cost analysis with those of the rest of the study suggests that management of ataxia at a SAC can lead to better care and improved patient satisfaction, with a higher cost for treatment in Germany. If we put these results in the larger perspective of this study, looking at the health costs in the UK and Italy for similar services, mean costs per patient are highest in Germany and lower in Italy and the UK [18]. One explanation was the differences in overall health expenditure per capita between the three countries with mean overall spend on health per capita in Germany being the fourth highest across all OECD (Organisation for Economic Co-operation and Development) countries. This should be taken into account when considering how to influence policy for better treatment and care for people with ataxia in Germany.

### 4.4 Limitations of the study

The recruitment for this study in Germany was made through both channels of patient advocacy groups membership and clinicians in SACs who have been involved in the dissemination of the survey. The additional recruitment through clinicians at SACs was to increase the number of participants, and this may have created a bias in the way they answered the questionnaires compared to the people recruited independently of the SACs. This group of people received the form and the instructions from healthcare professionals in SACs on how to complete the survey, and the patients completed the form themselves. A further limitation is that the number of participants who responded to the questions on health service use in the survey was low, which precluded statistical analyses of differences between groups. This was particularly the case when we investigated differences in participants stratified by the presence of comorbidities and the number of symptoms associated with ataxia.

### 4.5 Ways forward to improve ataxia treatment and care delivered

We advocate a system in which each patient has the right to choose the treating healthcare professional in an unbiased fashion, including referral to University Hospitals where the SACs are [10]. SACs in Germany don't have the prerogative to coordinate referrals to other relevant HCPs, or to communicate with social care professionals. Indeed, patients did not see any difference between SAC and non-SAC for these activities. Therefore, we recommend having this relevant role incorporated in the German SACs for the management of these complex diseases. This has been highly valued by the patients in the UK as demonstrated in the results of the same survey [21]. There is a significant number of SACs in Germany and they are members of the ERN-RNDs or the German Reference Network for Rare Neurological Diseases. On the healthcare system level, it is instrumental to explore ways to improve SAC access across the country, for example embedding within the healthcare system clear patient referrals to specialist centres. We believe that SACs should follow the UK model delivering the coordinated care and establishing more MDT clinics. In addition, the development of telemedicine should be advocated for ataxia patients [26], linking SAC with patients and primary care services to ensure a sustainable and personalised care plan for these patients is rolled out near their home. The establishment of medical centres for adult patients with disabilities (MZEB) in Germany and the reimbursement of the respective MDT service and care coordination are good steps forward [26]. These MZEBs should be accessible to all ataxia patients.

Finally, one way to optimize care for people with ataxia would be the establishment of a network-based healthcare model similarly to the Dutch ParkinsonNet for people with Parkinson's disease (PD) [27]. The aim of ParkinsonNet is to create networks of healthcare professionals and people living with PD, they train and educate healthcare professionals and make them experts in PD. This model could be implemented via the ERN-RND network [28] throughout Europe in order to establish a European cross-border AtaxiaNet. Ideally these changes should be supported by a European cross-border healthcare framework facilitating health equity.

## 5 Conclusion

With the perspective to level access to the best and adapted care for rare diseases between EU countries, this study brings evidence that having specialist centres for the ataxias is the crucial first step in order to ensure that people are offered the care they need. The survey results highlight the relevance of patients' voice to develop further the service based on the patients' needs. The SAC services that include patients' feedback have been pioneered in the UK [8,9] and has been proved effective in modifying the healthcare pathway for ataxia that has become patient centred. This model can be implemented in Germany to improve even further the ataxia patients’ management. A step forward would be the establishment of ERN network specifically for ataxia similarly to the Dutch PD network.

## CRediT authorship contribution statement

**Julie Vallortigara:** Writing – review & editing, Writing – original draft, Validation, Software, Resources, Project administration, Methodology, Investigation, Formal analysis, Data curation. **Julie Greenfield:** Writing – review & editing, Validation, Methodology, Conceptualization. **Barry Hunt:** Writing – review & editing, Validation, Methodology, Conceptualization. **Holm Graessner:** Writing – review & editing, Validation, Resources, Data curation. **Carola Reinhard:** Writing – review & editing, Validation, Resources, Data curation. **Andreas Nadke:** Validation, Resources, Data curation. **Bart-Jan Schumman:** Validation, Resources, Data curation. **Deborah Hoffman:** Methodology, Funding acquisition, Conceptualization. **Steve Morris:** Writing – review & editing, Writing – original draft, Validation, Software, Methodology, Investigation, Formal analysis. **Paola Giunti:** Writing – review & editing, Validation, Supervision, Resources, Project administration, Methodology, Funding acquisition, Conceptualization.

## Data statement

The data that support the findings of this study are available from the corresponding author on reasonable request.

## Organisation and financing of the study

This study is supported by the 10.13039/501100022661European Brain Council (EBC), and receives financial support from 10.13039/100008373Takeda Pharmaceutical Company Ltd and Reata Pharmaceutical Inc. It is being conducted by University College London, with the support of the patient organisations Deutsche Heredo Ataxie Gesellschaft (DHAG), Friedreich Ataxie Förderverein (FA), Ataxia UK and the European Reference Network for Rare Neurological Diseases (ERN-RND).

## Funding

This study was funded by the European Brain Council (grant code: 551578) and Reata Pharmaceuticals (grant code: NA). PG received funding from the MRC (grant code: MR/N028767/1).

## Declaration of competing interest

The authors declare that they have no known competing financial interests or personal relationships that could have appeared to influence the work reported in this paper.

## References

[1] Ashizawa T., Xia G. (2016). Ataxia. Continuum (Minneap Minn).

[2] Buckley E., Mazzà C., McNeill A. (2018). A systematic review of the gait characteristics associated with Cerebellar Ataxia. Gait Posture.

[3] Pilotto F., Saxena S. (2018). Epidemiology of inherited cerebellar ataxias and challenges in clinical research. Clin. Transl. Neurosci..

[4] Scott S.S.O., Pedroso J.L., Barsottini O.G.P., França-Junior M.C., Braga-Neto P. (2020 Oct 15). Natural history and epidemiology of the spinocerebellar ataxias: insights from the first description to nowadays. J. Neurol. Sci..

[5] Tranchant C., Anheim M. (2016 Aug-Sep). Movement disorders in mitochondrial diseases. Rev. Neurol. (Paris).

[6] Cossée M., Schmitt M., Campuzano V., Reutenauer L., Moutou C., Mandel J.L., Koenig M. (1997). Evolution of the Friedreich's ataxia trinucleotide repeat expansion: founder effect and permutations. Proc. Natl. Acad. Sci. U. S. A..

[7] Vankan P. (2013 Aug). Prevalence gradients of Friedreich's ataxia and R1b haplotype in Europe co-localize, suggesting a common Palaeolithic origin in the Franco-Cantabrian ice age refuge. J. Neurochem..

[8] Greenfield J., Treacy C., Giunti P. (2006). Centres of excellence for the care of people with progressive ataxias. Br. J. Nurs..

[9] Greenfield J J., Treacy C., Giunti P. (2008). Centres of excellence for the care of people with progressive ataxias. Br. J. Neurosci. Nurs..

[10] De Silva R R., Greenfield J., Cook A., Bonney H., Vallortigara J., Hunt B., Giunti P. (2019). Guidelines on the diagnosis and management of the progressive ataxias. Orphanet J. Rare Dis..

[11] Corben L.A., Lynch D., Pandolfo M., Schulz J.B., Delatycki M.B. (2014). Clinical management guidelines writing group, consensus clinical management guidelines for Friedreich ataxia. Orphanet J. Rare Dis..

[12] Abeti R., Baccaro A., Esteras N., Giunti P. (2018 Jul 17). Novel Nrf2-inducer prevents mitochondrial defects and oxidative stress in Friedreich's ataxia models. Front. Cell. Neurosci..

[13] Pilotto F., Chellapandi D.M., Puccio H. (2024). Omaveloxolone: a groundbreaking milestone as the first FDA-approved drug for Friedreich ataxia. Trends Mol. Med..

[14] European Medicine Agency, Science Medicines Health, Skyclarys. https://www.ema.europa.eu/en/medicines/human/EPAR/skyclarys.

[15] Giunti P., Morris S., Relja M., Pastores G., Quoidbach V. (2019 Apr). Toward earlier diagnosis and treatment of rare neurological disorders: the value of coordinated care and specialist centers. Croat. Med. J..

[16] DRN-RND: German Reference Network for Rare Neurological Diseases. https://se-atlas.de/map/drn/Deutsches%20Referenznetzwerk%20f%C3%BCr%20Seltene%20Neurologische%20Erkrankungen?ln=en_EN.

[17] Vallortigara J., Greenfield J., Hunt B., Hoffman D., Reinhard C., Graessner H., Federico A., Quoidbach V., Morris S., Giunti P. (2023 Oct 17). Patient pathways for rare diseases in Europe: ataxia as an example. Orphanet J. Rare Dis..

[18] Morris S., Vallortigara J., Greenfield J., Hunt B., Hoffman D., Reinhard C., Graessner H., Federico A., Quoidbach V., Giunti P. (2023 Dec 7). Impact of specialist ataxia centres on health service resource utilisation and costs across Europe: cross-sectional survey. Orphanet J. Rare Dis..

[19] Pöhlmann J., Norrbacka K., Boye K.S., Valentine W.J., Sapin H. (2020). Costs and where to find them: identifying unit costs for health economic evaluations of diabetes in France, Germany and Italy. Eur. J. Health Econ..

[20] StataCorp (2017).

[21] Vallortigara J., Greenfield J., Hunt B., Hoffman D., Booth S., Morris S., Giunti P. (2024 Sep 5). Comparison of specialist ataxia centres with non-specialist services in terms of treatment, care, health services resource utilisation and costs in the UK using patient-reported data. BMJ Open.

[22] Manto M., Gandini J., Feil K., Strupp M. (2020 Feb). Cerebellar ataxias: an update. Curr. Opin. Neurol..

[23] Pakter P. (2024). Rare disease care in Europe – gaping unmet needs. Rare.

[24] National Ataxia Foundation, Ataxia Centers of Excellence. https://www.ataxia.org/ace/.

[25] Giunti P., Greenfield J., Stevenson A.J., Parkinson M.H., Hartmann J.L., Sandtmann R., Piercy J., O'Hara J., Casas L.R., Smith F.M. (2013 Feb 28). Impact of Friedreich's Ataxia on health-care resource utilization in the United Kingdom and Germany. Orphanet J. Rare Dis..

[26] Reinhard C., Bachoud-Lévi A.C., Bäumer T., Bertini E., Brunelle A., Buizer A.I., Federico A., Gasser T., Groeschel S., Hermanns S., Klockgether T., Krägeloh-Mann I., Landwehrmeyer G.B., Leber I., Macaya A., Mariotti C., Meissner W.G., Molnar M.J., Nonnekes J., Ortigoza Escobar J.D., Pérez Dueñas B., Renna Linton L., Schöls L., Schuele R., Tijssen M.A.J., Vandenberghe R., Volkmer A., Wolf N.I., Graessner H. (2021 Jan 14). The European reference network for rare neurological diseases. Front. Neurol..

[27] ParkinsonNet. https://www.parkinsonnet.com/.

[28] European Reference Network, Neurological Diseases (ERN-RND). https://www.ern-rnd.eu/.

