# Supplementary material for: Comparison of specialist ataxia centres with non-specialist services in terms of care access and organisation, health services resource utilisation and costs in Germany using patient-reported data
Source: Heliyon. 2025 Jan 29;11(3):e42121. doi: 10.1016/j.heliyon.2025.e42121 (PMC11849067; doi:10.1016/j.heliyon.2025.e42121)
Supplement: Multimedia component 1 [file mmc1.docx]

**APPENDIX 1: Ataxia Care Pathways – Germany Patient Survey**

Thank you for taking the time to complete the survey below. Please take the time to read the survey information carefully and feel free to discuss it with others, such as family or friends.

With this survey we would like to find out what experiences you have as ataxia patients in Germany in order to better understand the care situation of ataxia patients in Germany and to find out what role ataxia specialist centres play in this.

If you have been diagnosed with ataxia at a very young age, you are welcome to discuss the survey with your parents. If you, as a parent, caregiver, and caregiver, are helping to complete the survey, you should reflect the patient's opinion, not your own.

**Information on data protection:**

All data is collected anonymously, and no personal information is requested, so that it is not possible to draw conclusions about your person. Your answers will be summarized with the answers of the other study participants before the analysis. All data is stored password-protected so that only authorized persons have access to it. All data will be destroyed 6 months after completion of the data evaluation.

If you would like further information about the study or a copy of the study results, please contact Dr. Carola Reinhard, project manager of the ERN-RND, at Carola.Reinhard@med.uni-tuebingen.de, phone: 07071/29-72191.

**Organisation and financing of the study:**

This study is supported by the European Brain Council (EBC), and receives financial support from Takeda Pharmaceutical Company Ltd and Reata Pharmaceutical Inc. It is being conducted by University College London, with the support of the patient organisations Deutsche Heredo Ataxie Gesellschaft (DHAG), Friedreich Ataxie Förderverein (FA), Ataxia UK and the European Reference Network for Rare Neurological Diseases (ERN-RND).

**Contact details:**

If you have any further questions about the study or the questionnaire, please contact:

Contact for DHAG: Andreas Nadke, Mail: andreas.nadke@outlook.de, Phone: 0711/5504644, Address : Hofener Strasse 76, 70372 Stuttgart

Contact for the FA: Jan-Bart Schumann, Mail: vorstand@friedreichataxie.de, Phone: 08121/4100723, Address: Am Heckenacker 9a, 85652 Pliening

**Instructions for completing the questionnaire**

Please answer all questions by ticking the appropriate box or by writing your answer in the respective text field.

Please send the completed questionnaire in the enclosed stamped envelope to:

Deutsche Heredo Ataxie Gesellschaft, Hofener Strasse 76, 70372 Stuttgart

**Definitions and terms used below:**

General ataxia diagnosis: The time you were first diagnosed with ataxia

Specific diagnosis of ataxia: The time when you were told that you have a certain form of ataxia, such as spinocerebellar ataxia type 1. You may not have received a specific diagnosis of ataxia at the time of the survey.

For the purposes of this survey, idiopathic ataxias and cerebellar ataxias (unclear cause) are not considered specific diagnoses.

Specialist ataxia centre: A centre that specialises in the care of ataxia patients. In the context of this survey, the following institutions are included:

- University Hospital Munich

- University Hospital Tübingen

- University Hospital Bonn

- University Hospital Schleswig-Holstein (Lübeck)

- University Hospital Essen

- University Hospital Aachen

- Charité, University Hospital Berlin

- University Hospital Düsseldorf

- University Hospital Magdeburg

For the purposes of this survey, please only indicate that you are/have been treated at a specialist ataxia centre if it is one of the centres mentioned above.

General Department of Neurology: Neurological department in a clinic that does not belong to the above-mentioned ataxia specialty centres.

Primary Care: Medical care by a general practitioner and other established healthcare professionals

Inpatient care: Care in the hospital "overnight"

Outpatient care: Care in hospital without inpatient admission, e.g. for examinations or outpatient operations

Clinic with multidisciplinary teams: Multidisciplinary teams include more than one of the following health professions as part of the same service (within a clinic): neurologist, geneticist, physiotherapist, occupational therapist, speech therapist

**Clinic: Contact**

Friedrich Baur Institute at the University Hospital of Munich: Prof. Dr. Thomas Klopstock, Dr. Ivan Karin

Unikinik Tübingen: Prof. Dr. Ludger Schöls, Prof. Dr. Matthis Synofzik

University Hospital Bonn: Prof. Dr. Thomas Klockgether

University Hospital Schlesweg-Holstein: Prof. Dr. med. A. Münchau, PD Dr. med. Y. Hellenbroich, Prof. Dr. med. N. Brüggemann

University Hospital Essen: Prof. Dr. med. D. Timmann-Braun

University Hospital RWTH Aachen: Univ.-Prof. Dr. med. Kathrin Reetz, Dr. med. Florian Holtbernd

Charité University Medicine Berlin: Prof. Dr. med. Stephan Brandt, Dr. Sarah Doss, Maria Rönnefarth

University Hospital Düsseldorf: PD Dr. med. Martina Minnerop

University Department of Neurology Magdeburg: Prof. Dr. med. Stefan Vielhaber

**Instructions for completing the questionnaire**

It is not absolutely necessary to answer one question to get to the next question.

Questions 1 and 3 are screening questions, question 12 asks about your ataxia diagnosis. If you have to answer "No" or "Unclear" to any of these questions, please complete the survey.

**SURVEY**

**1) General/demographic issues**

Question 1 – mandatory to participate in the rest of the survey

I confirm that I (or the person on whose behalf I am participating in the survey) am at least 16 years old, live in Germany and would like to participate in the rest of the survey

Please tick:

- Yes

- No (please stop completing the questionnaire)

Question 2

Please indicate whether you yourself suffer from ataxia, or whether you are completing the survey on behalf of another person, representing that person's view with ataxia.

Please tick:

- Yes, I am an ataxia patient

- No, I am filling out the survey on behalf of another person suffering from ataxia

Question 3

Have you, or the patients on whose behalf you complete the survey, received a diagnosis of ataxia from a doctor?

Please tick

- Yes

- No (please stop completing the questionnaire)

- Unclear (please stop completing the questionnaire)

Question 4

How old are you?

Please tick

- 16-29

- 30-59

- 60-80

- 80+

Question 5

Which of the following terms best describes your gender?

- Female

- Male

- Diverse

Question 6

Do you suffer from other diseases that are not related to ataxia?

Please tick (multiple answers possible)

- Diabetes mellitus

- Kidney disease

- Krebs

- HIV or AIDS

- Chronic liver disease

- Heart failure

- Heart attack

- Chronic obstructive pulmonary disease (COPD)

- Peripheral vascular diseases

- Stroke or transient ischemic attack

- Dementia, e.g. Alzheimer's disease

- Psychiatric illness (depression or other)

- Hemiplegia (paralysis of one side of the body)

- Rheumatism or connective tissue disease

- Gastric ulcers

- None

- Other (please specify in the text box below)

- I don't know

Text box

Question 7

Do you live alone?

Please tick (multiple answers possible)

- Yes, I live alone

- No, I live with a partner or with other family members, I do not need support that significantly limits the lives of these people

- No, I live with a professional caregiver

- No, I live with a caregiver from the family/circle of friends

Question 8

In which federal state do you live?

Please tick

- Hamburg

- Bremen

- Berlin

- Lower Saxony

- Schleswig-Holstein

- North Rhine-Westphalia

- Hesse

- Rhineland-Palatinate

- Brandenburg

- Mecklenburg-Western Pomerania

- Thuringia

- Saxony

- Saxony-Anhalt

- Baden-Württemberg

- Saarland

- Bavaria

- Other, please specify in the text box below

- I don't know

Question 9

Which of the following best describes how ataxia affects your mobility?

Please tick

- No functional limitation

- Mild impairment, I can walk and run without restriction

- Moderate impairment, I can't run, I can walk a limited distance without aids

- I need a cane to walk

- In the house I need two sticks / a walker, outside the house I need a wheelchair

- I can't walk and need a wheelchair, but otherwise I'm independent of help

- I can't walk and need a wheelchair, depend on help

- I am bedridden

Question 10

How does ataxia affect your professional life?

Please tick (multiple answers possible)

- I am still in training

- I am employed

- I am in retirement pension

- Because of my ataxia, I never worked

- Due to my ataxia, I am in early retirement. Please indicate the age at retirement in the text box below

- Due to my ataxia, I changed professions

- Due to my ataxia, I reduced my working hours

- Because of my ataxia, I get social assistance

- Due to my ataxia, I received financial support from my family.

- Due to my ataxia, I was on sick leave. Please enter the number of days of attendance in the text box below.

- Other. Please specify in the text box below.

Text box

Question 11

Which doctor is currently responsible for the care of your ataxia?

Please tick

- Family doctor

- Established neurologist/neurologist in a general neurological department of a hospital

- Neurologist in a specialist ataxia centre

- Other doctor, please specify in the text box below

- I do not know.

Text box

**2) Diagnosis**

Question 12

What ataxia do you have?

Please tick

- Friedreich Ataxia

- Hereditary cerebellar ataxia (SCAs, e.g. SCA1, SCA2, SCA3)

- Episodic ataxia

- Other. Please specify in the text box below

- Unclear

- None. Please stop completing the questionnaire.

Text box

Question 13

From whom did you first receive the general diagnosis of ataxia?

Please tick

- Family doctor

- Neurologist

- Physiotherapist

- Human geneticist

- Other. Please specify in the text box below.

- I don't know

Text box

Question 14

When did this person make this first general diagnosis of ataxia? Please specify month (if known) and year in the text box.

Text box

- I do not know

Question 15

Has your specific ataxia diagnosis been confirmed? E.g. by a genetic test?

Please tick

- Yes, I have a genetically confirmed diagnosis. E.g. Friedreich ataxia, spinocerebellar ataxia type 1, 2, 3 etc. (SCA1, SCA2, SCA3 etc.). Please mention the specific diagnosis in the text box below.

- Yes, I have another confirmation of my diagnosis, e.g. gluten ataxia. Please mention the specific diagnosis in the text box below.

- No

- I don't know

Text box

Question 16

Where has your specific ataxia diagnosis been confirmed?

Please tick

- In a specialist ataxia centre with a resident neurologist

- In a hospital with a general neurological department

- Other. Please specify in the text box below

- I don't know

Text box

Question 17

How long did it take from the time you first visited a neurologist to receive a specific diagnosis of ataxia (e.g. Friedreich ataxia, spinocerebellar ataxia type 1, etc.)?

Please tick

- I received the specific ataxia diagnosis when I first visited a neurologist for my ataxia

- Up to 6 months

- Between 6 months and 1 year

- Between 1 and 2 years

- Between 2 and 5 years

- More than 5 years

- I have not received a specific diagnosis of ataxia (please continue with question 19)

- I don't know

Question 18

How many times have you been told another diagnosis by your neurologist, e.g. multiple sclerosis, dyspraxia, apraxia, ear problems, dizziness, etc., before you have received the specific diagnosis of ataxia?

Please tick

- No

- Once

- Twice

- Three times

- Four times

- Five times or more

- I don't know.

Question 19

How limited were you in the activities of daily living by your ataxia at the time you received your first general ataxia diagnosis?

Please tick

- My ataxia didn't limit me.

- My ataxia caused problems every now and then.

- My ataxia caused frequent problems and limited me in my activities.

- My ataxia caused permanent problems and limited me most of the time.

- I don't know

Question 20

How much does ataxia restrict you at the present time?

Please tick

- My ataxia does not limit me.

- My ataxia causes problems every now and then.

- My ataxia causes frequent problems and restricts me in my activities.

- My ataxia causes permanent problems and limits me most of the time.

- I don't know

Question 21

If you have a genetic diagnosis, have you discussed its impact on insurance aspects and family planning with a medical professional?

Please tick

- Yes

- No

- Not specified

- I don't know

**3) Transfers and appointments**

Question 22

How long did it take after you first sought medical advice (e.g. from your family doctor) about your ataxia symptoms before you were referred to a neurologist?

Please tick

- Up to 6 months

- 6-12 months

- 1-2 years

- 2-5 years

- More than 5 years

- I don't know

Question 23

What kind of neurologist were you referred to?

- Neurologist in a hospital with a general neurological department

- Neurologist in a specialist ataxia centre

- Other. Please specify in the text box below.

- I don't know

Text box

Question 24

How many of the following doctor/clinic visits did you have before the first (general) ataxia diagnosis?

Please specify one per option.

Doctor's visits Family doctor

- None

- 1

- 2-3

- 4-5

- More than 5

- I don't know

Doctor's visits to a resident neurologist / outpatient visit to a neurologist in a hospital with a general neurological department

- None

- 1

- 2-3

- 4-5

- More than 5

- I don't know

Visits to an Specialist Ataxia Centre (SAC)

- None

- 1

- 2-3

- 4-5

- More than 5

- I don't know

Number of hospitalisations

- None

- 1

- 2-3

- 4-5

- More than 5

- I don't know

Number of nights of inpatient stay

- None

- 1

- 2-3

- 4-5

- More than 5

- I don't know

Number of emergency room visits

- None

- 1

- 2-3

- 4-5

- More than 5

- I don't know

Number of physiotherapy treatments

- None

- 1

- 2-3

- 4-5

- More than 5

- I don't know

Number of speech therapy treatments

- None

- 1

- 2-3

- 4-5

- More than 5

- I don't know

Number of occupational therapy treatments

- None

- 1

- 2-3

- 4-5

- More than 5

- I don't know

Number of visits to other specialists (e.g. ophthalmologist, ear, nose and throat doctor). Please specify the specialisation in the text box below

- None

- 1

- 2-3

- 4-5

- More than 5

- I don't know

Text box

How many of the following doctor/clinic visits did you have before the specific ataxia diagnosis?

Please specify one per option:

Doctor's visits Family doctor

- None

- 1

- 2-3

- 4-5

- More than 5

- I don't know

Doctor's visits to a resident neurologist / outpatient visit to a neurologist in a hospital with a general neurological department

- None

- 1

- 2-3

- 4-5

- More than 5

- I don't know

- Visits to an Ataxia Specialist Centre

- None

- 1

- 2-3

- 4-5

- More than 5

- I don't know

Number of hospitalisations

- None

- 1

- 2-3

- 4-5

- More than 5

- I don't know

Number of nights of inpatient stay

- None

- 1

- 2-3

- 4-5

- More than 5

- I don't know

Number of emergency room visits

- None

- 1

- 2-3

- 4-5

- More than 5

- I don't know

Number of physiotherapy treatments

- None

- 1

- 2-3

- 4-5

- More than 5

- I don't know

Number of speech therapy treatments

- None

- 1

- 2-3

- 4-5

- More than 5

- I don't know

Number of occupational therapy treatments

- None

- 1

- 2-3

- 4-5

- More than 5

- I don't know

Number of visits to other specialists (e.g. ophthalmologist, ear, nose and throat doctor). Please specify the specialization in the text box below

- None

- 1

- 2-3

- 4-5

- More than 5

- I don't know

Text box

Question 24

How many of the following doctor/clinic visits have you had in the past 12 months due to your ataxia?

Please specify one per option.

Doctor's visits Family doctor

- None

- 1

- 2-3

- 4-5

- More than 5

- I don't know

Doctor's visits to a resident neurologist / outpatient visits to a neurologist in a hospital with a general neurological department

- No

- 1

- 2-3

- 4-5

- More than 5

- I don't know

Visits to an Specialist Ataxia Centre

- None

- 1

- 2-3

- 4-5

- More than 5

- I don't know

Number of hospitalisations

- None

- 1

- 2-3

- 4-5

- More than 5

- I don't know

Number of nights of inpatient stay

- No

- 1

- 2-3

- 4-5

- More than 5

- I don't know

Number of emergency room visits

- None

- 1

- 2-3

- 4-5

- More than 5

- I don't know

Number of physiotherapy treatments

- None

- 1

- 2-3

- 4-5

- More than 5

- I don't know

Number of speech therapy treatments

- None

- 1

- 2-3

- 4-5

- More than 5

- I don't know

Number of occupational therapy treatments

- None

- 1

- 2-3

- 4-5

- More than 5

- I don't know

Number of visits to other specialists (e.g. ophthalmologist, ear, nose and throat doctor). Please specify the specialization in the text box below

- None

- 1

- 2-3

- 4-5

- More than 5

- I don't know

Text box

Question 25

Have you ever been referred to one of the specialist ataxia centres/have you ever visited one of these centres?

Please tick

- Yes, I am currently being treated in a specialist ataxia centre.

- Yes, I have been treated in a specialist ataxia centre in the past, but I am currently no longer being treated there.

- No. Please continue with question 35.

- I don't know. Please continue with question 35.

Question 26

Have you already been treated by a resident neurologist at a hospital with a general neurological department before you were referred to a specialist ataxia centre?

Please tick

- Yes

- No

- I don't know

Question 27

When were you referred to a specialised ataxia center? Please specify month (if known) and year in the text box.

Text box

- I don't know

Question 28

By whom were you transferred?

Please tick

- Family doctor

- Established neurologist/neurologist working in a hospital with a general neurological department

- Other. Please specify in the text box below.

- I don't know

Text box

Question 29

If you have been treated by a resident neurologist working in a hospital with a general neurological department before visiting a specialist ataxia centre, you will find that the care in a specialist centre for ataxia is better than the previous treatment.

- Yes

- No

- Not specified

- I don't know

Question 30

Please compare your experience at the specialist ataxia centre with that of a resident neurologist working in a hospital with a general neurological department.

Please tick the box in each row that best characterizes your experience. Please answer this question only if you have experience with care in an specialist ataxia centre as well as outside.

Much better in a specialist ataxia centre / Better in an specialist ataxia centre / Neither better nor worse in an specialist ataxia centre / Worse in an specialist ataxia centre / Much worse in an specialist ataxia centre

A good understanding of my state of health

Allows me to better manage my illness

Gives practical tips on how I can live with my illness

Gives medical advice for better management of my symptoms

Coordinates referrals to other specialists

Offered me to participate in research projects

Improved communication between health and social care professionals

Question 31

Please indicate the reason why you are no longer being treated in a Specialist Ataxia Centre.

Please tick

- Accessibility issues

- I didn't think it was necessary

- I was no longer transferred there

- Local supply is equivalent

- I am not able to take time off to visit the Specialist Ataxia Centre

- Other. Please specify in the text box below

- I don't know

Text box

Question 32

How much travel time do you need/did you need to visit an Specialist Ataxia Centre (one way)?

Please tick

- Less than 1 hour

- 1-2 hours

- 2-3 hours

- 3-4 hours

- More than 4 hours

- I don't know

Question 33

Which means of transport do you mainly/have you mainly used to visit the Specialist Ataxia Centre?

Please tick

- Ambulance service

- Car

- Bus

- Course

- Taxi

- Walk

- Other. Please specify in the text box below.

- I don't know

Text box

Question 34

Do you need to stay overnight on site/did you have to stay overnight on site to visit a Specialist Ataxia Centre?

Please tick

- Yes, in accommodation provided by the hospital

- Yes, in an accommodation that I paid for privately

- No

- I don't know

Question 35

What are the reasons why you have never been referred to an Specialist Ataxia Centre /Never visited an Specialist Ataxia Centre?

Please tick

- My current supply situation is sufficient.

- I asked for referral to a special centre for ataxia, but the physician refuse it.

- The Specialist Ataxia Centre is too far away to travel to.

- I did not want to be referred to a Specialist Ataxia Centre.

- A referral was not offered.

- Other. Please specify in the text box below.

- Not specified

- I don't know

Text box

Question 36

How long do you need to visit your treating neurologist/neurologist in a hospital with a general neurological department (one way)?

Please tick

- Less than 1 hour

- 1-2 hours

- 2-3 hours

- 3-4 hours

- More than 4 hours

- Not specified

- I don't know

**4) Satisfaction with care**

Question 37

Have you ever been seen by a multidisciplinary team (MDT) including at a non-specialist hospital?

- Yes

- No. Continue to question 42.

- I don't know. Continue to question 42.

Question 38

Who referred you to the Multidisciplinary Team?

- Neurologist in a Specialist Ataxia Centre

- Neurologist in a hospital with a general neurological department

- Other. Please specify in the text box below.

Text box

Question 39

When were you referred to a multidisciplinary team?

Please specify month (if known) and year in the text box.

Text box

- I don't know

Question 40

If you were cared for by a multidisciplinary team, how effective would you consider that care?

Please tick

- Very effective (Continue to question 42)

- Effective (continue to question 42)

- Neither effective nor ineffective (Continue to question 42)

- Ineffective

- Very ineffective

Question 41

What were the reasons why you found the care provided by a multidisciplinary team to be ineffective?

Please tick (multiple answers possible)

- No referral to a local primary care team, e.g. a local physiotherapist

- No referral to a specialist at a specialist centre, e.g. a specialist urologist at a university hospital

- Referral to a specialist at a specialist centre was not effective

- The multidisciplinary team did not understand my needs

- No treatment available, even after consultation with the Multidisciplinary Team

- Other. Please specify in the text box below.

- I don't know

Questions 42

Please rate your agreement with the following 12 statements

Please make one rating per statement.

Strongly Agree / Agree / Neither agree or disagree / Disagree / Strongly disagree / Not specified

My family doctor understands how to take care of my ataxia.

My family doctor knows the available treatment options for my ataxia.

My resident neurologist/neurologist in a hospital with a general neurological ward understands how to care for my ataxia.

My resident neurologist/neurologist in a hospital with a general neurological ward knows the available treatment options for my ataxia.

My neurologist at the Specialist Ataxia Centre understands how to treat my ataxia.

My neurologist at the Specialist Ataxia Centre knows the available treatment options for my ataxia.

Emergency medical staff understand how my ataxia can affect treatment.

Please indicate to what extent you agree with the following statement:

The care I received in the emergency room could have been better, for example, if the medical staff had known more about my illness, if patients had a card with important information about the disease that you could show to the medical staff.

Question 43

Have you ever been referred for treatment for any of the following ataxia symptoms?

Please answer the questions by ticking the box in the row next to each symptom.

A – Yes, I have suffered from this symptom and have been referred for appropriate treatment.

B – No, I have suffered from this symptom but have not been referred for appropriate treatment.

C – I have never suffered from this symptom.

D – Don't know.

Symptom A B C D

Ache

Heart problems

Gastroenterological problems

Sexual dysfunction

Problems with hearing

Eye problem

Insomnia

Problems with the language

Depression

Other mental disorders

Fatigue

Other. Please specify in the text box below.

Text box

Question 44

Please list any symptoms you have suffered from in the past year and the treatment you have received for each symptom.

Please tick (multiple answers possible)

Dystonia: Uncontrolled and sometimes painful muscle movements that can lead to unnatural positions of individual parts of the body.

Stiffness of the legs or muscles – Cramps – Spasticity - Bladder Problem - Dystonia - Tremor/tremors

I suffered from this symptom

I have seen a family doctor to have the symptom treated

I went to another health service to have the problem treated

I visited a resident neurologist/neurologist in a hospital with a general neurological department to have the symptom treated

I visited a neurologist at a Specialist Ataxia Centre to have the symptom treated

I have received treatment for the symptom outside of the Specialist Ataxia Centre

I have received treatment for the symptom within the Specialist Ataxia Centre

I have not sought treatment for the symptom

I have not been offered treatment for the symptom

Question 45

If you have suffered from the following symptoms in the past year and have received treatment for the particular symptom, please indicate the form in which the treatment took place.

Please tick (multiple answers possible)

- Stiffness of the legs or muscles

o Drug therapy (oral or injection)

o Physiotherapy

o Occupational Therapy

o Alternative therapies (e.g. homeopathy, acupuncture)

o Exercise therapy (e.g. gym, Pilates)

o Other. Please specify in the text box below.

o I don't know

Question 46

If you have suffered from the following symptoms in the past year and have received treatment for the particular symptom, please indicate the form in which the treatment took place.

- Cramps

o Drug therapy (oral or injection)

o Physiotherapy

o Occupational Therapy

o Alternative therapies (e.g. homeopathy, acupuncture)

o Exercise therapy (e.g. gym, Pilates)

o Other. Please specify in the text box below.

o I don't know

Question 47

If you have suffered from the following symptoms in the past year and have received treatment for the particular symptom, please indicate the form in which the treatment took place.

- Spasticity

o Drug therapy (oral or injection)

o Physiotherapy

o Occupational Therapy

o Surgery

o Alternative therapies (e.g. homeopathy, acupuncture)

o Exercise therapy (e.g. gym, Pilates)

o Other. Please specify in the text box below.

o I don't know

Question 48

If you have suffered from the following symptoms in the past year and have received treatment for the particular symptom, please indicate the form in which the treatment took place.

- Bladder Problem

o Drug therapy (oral or injection)

o Catheterisation

o nerve Stimulation

o Exercise therapy (e.g. gym, Pilates)

o Other. Please specify in the text box below.

o I don't know

Question 49

If you have suffered from the following symptoms in the past year and have received treatment for the particular symptom, please indicate the form in which the treatment took place.

- Dystonia

o Drug therapy (oral or injection)

o Physiotherapy

o Occupational Therapy

o Exercise therapy (e.g. gym, Pilates)

o Other. Please specify in the text box below.

o I don't know

Question 50

If you have suffered from the following symptoms in the past year and have received treatment for the particular symptom, please indicate the form in which the treatment took place.

- Tremor/tremors

o Drug therapy (oral or injection)

o Occupational Therapy

o Exercise therapy (e.g. gym, Pilates)

o Other. Please specify in the text box below.

o I don't know

Question 51

If you have had one or more of the following symptoms in the past year and have received treatment for them, how do you rate the treatment of the symptoms?

Please answer the questions by ticking the box in the row next to each symptom.

Best it could be Very well Adequately Poorly Very poorly Not specified

Stiffness of the legs or muscles

Cramps

Spasticity

Bladder Problem

Dystonia

Tremor/tremors

Question 52

In summary, taking into account the above symptoms and all the other symptoms you suffer from due to your ataxia, do you feel that your symptoms are being managed well?

Please tick

- Best it could be

- Very well

- Adequately

- Poorly

- Very poorly

- Not specified

Question 53

Please estimate how well your care is tailored to your needs.

Please tick

- Best it could be

- Very well

- Adequately

- Poorly

- Very poorly

- Not specified

Question 54

What should be done to improve your care?

Please tick (multiple answers possible)

- More information about my disease

- More help to make me feel in control of my illness

- Knowing my specific diagnosis earlier

- Better management of my symptoms

- Better practical advice on how to live with my condition

- Improved access to therapies (e.g. physiotherapy, speech therapy, occupational therapy)

- More information on how to adapt my living environment to the needs of my illness

- Help with communication with my employer

- More information about the genetic cause of my disease and whether my children/grandchildren are at risk

- Continuation of the standard of care close to home if I am no longer able to visit an Specialist Ataxia Centre

- I am satisfied with my care and do not need any improvements

- Other. Please specify in the text box below.

- I don't know

Text box
