# Supplementary material for: Comparison of specialist ataxia centres with non-specialist services in terms of care access and organisation, health services resource utilisation and costs in Germany using patient-reported data
Source: Heliyon. 2025 Jan 29;11(3):e42121. doi: 10.1016/j.heliyon.2025.e42121 (PMC11849067; doi:10.1016/j.heliyon.2025.e42121)
Supplement: Multimedia component 2 [file mmc2.docx]

Figure 1: Time spent living with ataxia since first diagnosis was given by a Healthcare professional

1: 1-5 years; 2: 6-10 years; 3: 11-15 years; 4: 16-20 years; 5: 21-25 years; 6: 26-30 years; 7: 31-35 years; 8: 36-40 years.

Table 1: Level of mobility of the participants

| **Answer choices** | **Responses N (%)** |
| --- | --- |
| No functional restrictions | 4 (4) |
| Mild impairment, I can walk and run without restriction | 20 (20) |
| Moderate impairment, I cannot run, I can walk a limited distance without aids | 21 (21) |
| I need a stick to walk | 11 (11) |
| In the house I need two sticks / a rollator, outside I need a wheelchair | 19 (19) |
| I can’t walk and need a wheelchair, but otherwise I don’t need help | 1 (7) |
| I can’t walk and need a wheelchair, and I am dependent on help | 17 (17) |
| I am bedridden | 1 (1) |
| **Total** | 100 (100) |

Table 2: Burden of ataxia on people’s lives

| **Answer choices** | **Responses N (%)** |
| --- | --- |
| My ataxia doesn’t limit me | 4 (4.1) |
| My ataxia causes problems every now and then | 16 (16.5) |
| My ataxia causes frequent problems and limits my activities | 25 (25.8) |
| My ataxia causes permanent problems and restricts me most of the time | 53 (53.6) |
| I do not know | 0 (0) |
| **Total** | 97 (100) |

Table 3: Level of severity at the beginning of the pathway, when the first diagnosis was given
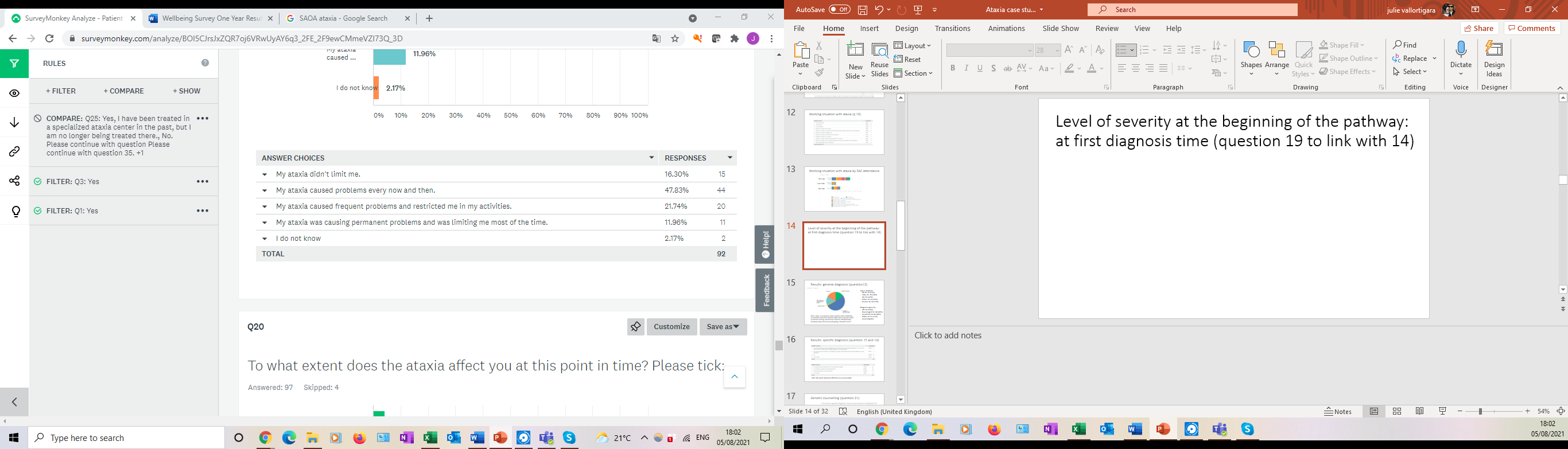


Table 4: Proportion of participants who received a confirmed diagnosis with genetic or other tests


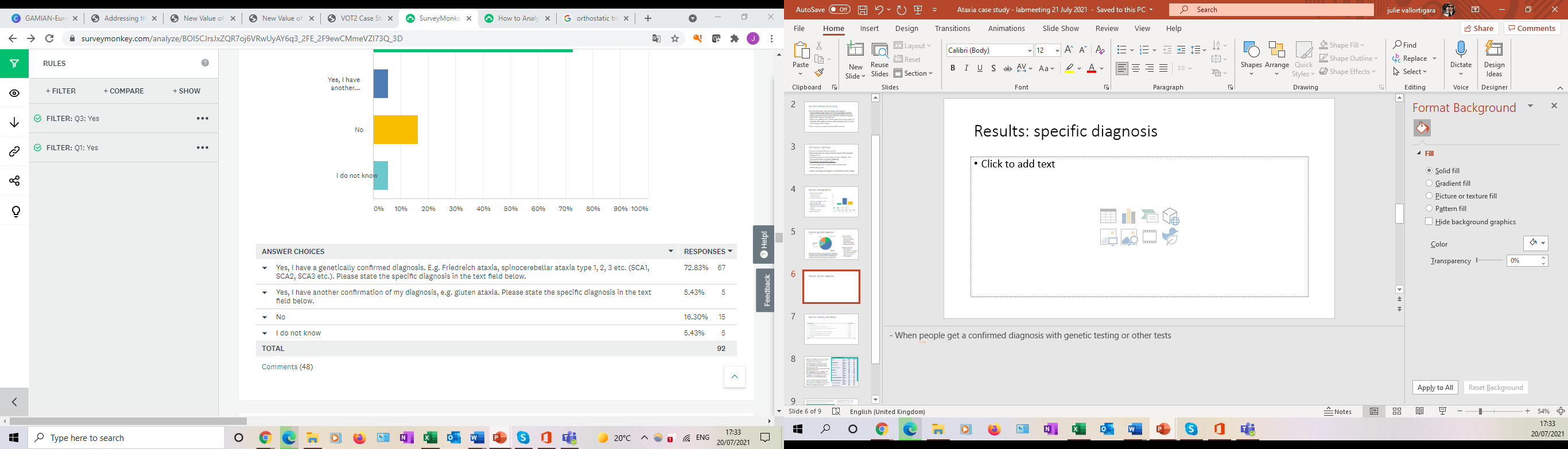


Table 5: Place where participants received their confirmed diagnosis of ataxia


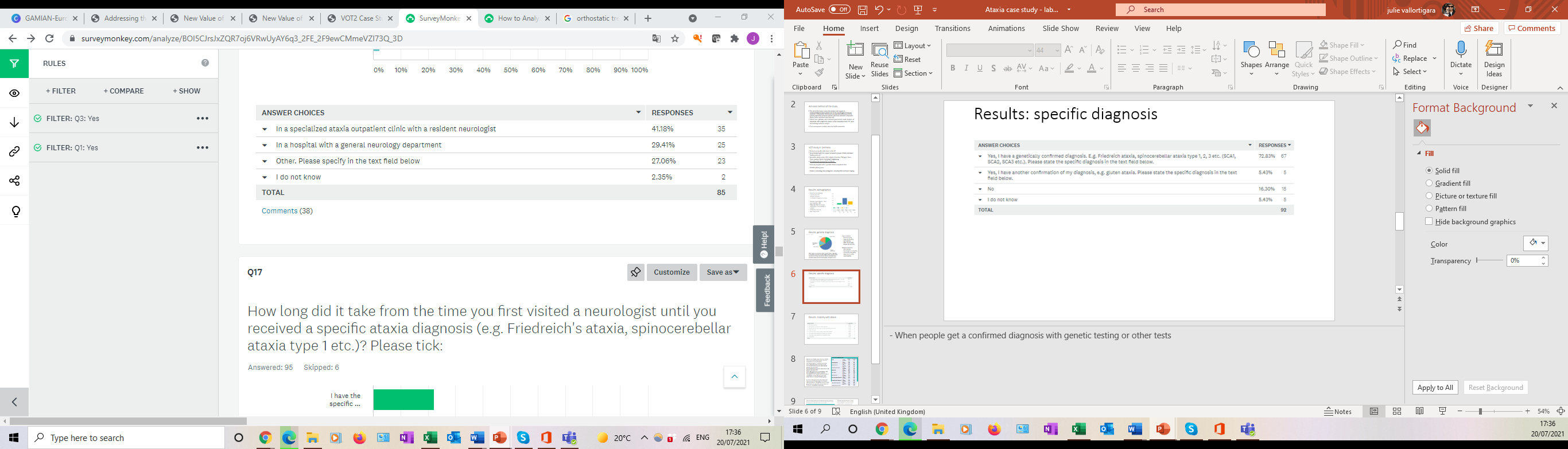


Figure 2: Confirmed diagnosis by SAC attendance


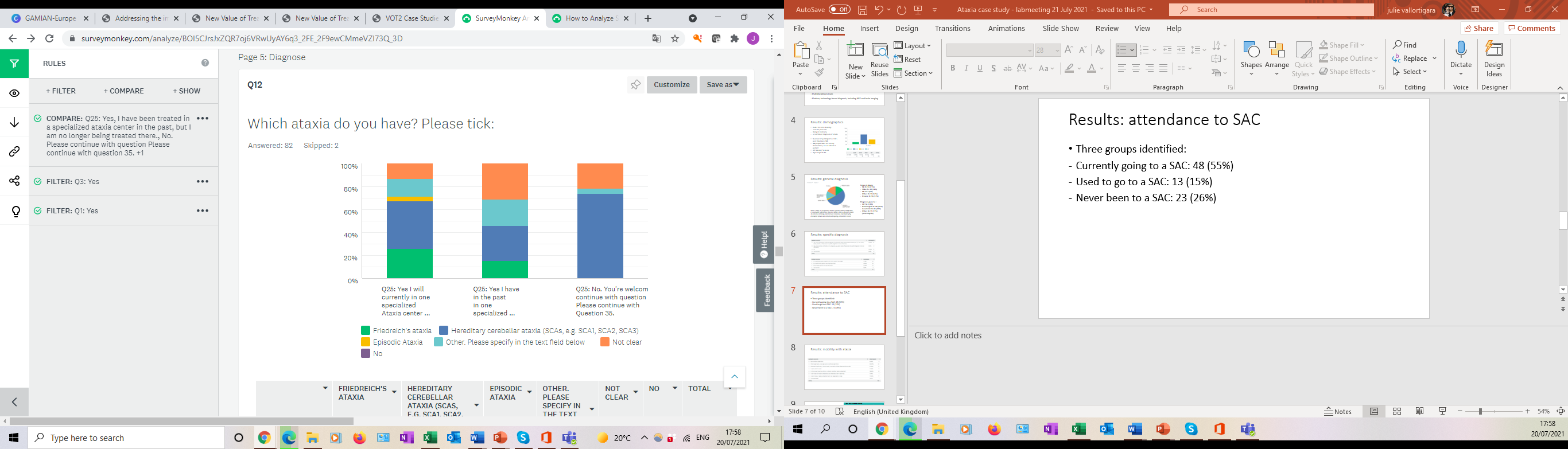


Figure 2 bis: Time between neurologist visit and specific diagnosis given by SAC attendance
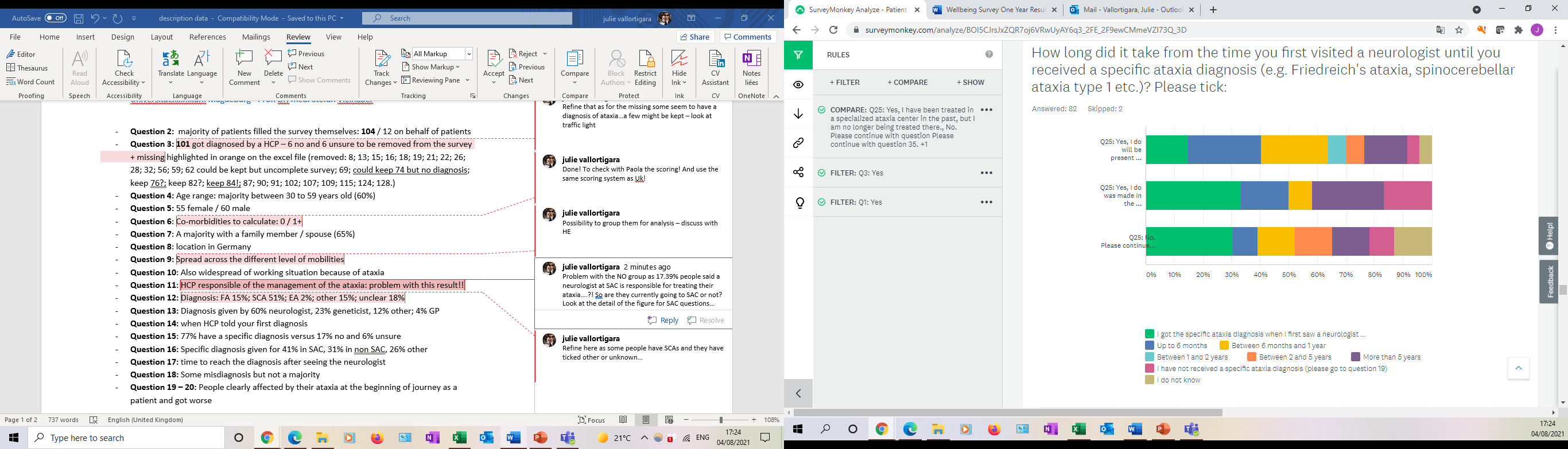


| **Answer choices** | **Responses N (%)** |
| --- | --- |
| Within 6 months | 42 (48.3) |
| Between 6 months and one year | 8 (9.2) |
| Between 1-2 years | 6 (6.9) |
| Between 2-5 years | 6 (6.9) |
| More than 5 years | 4 (4.6) |
| Unsure | 21 (24.1) |
| **Total** | **87 (100)** |

Table 6: Time spent between first medical advice on ataxia symptoms and referral to see a neurologist

Table 7: Where was the neurologist participants saw for their first referral


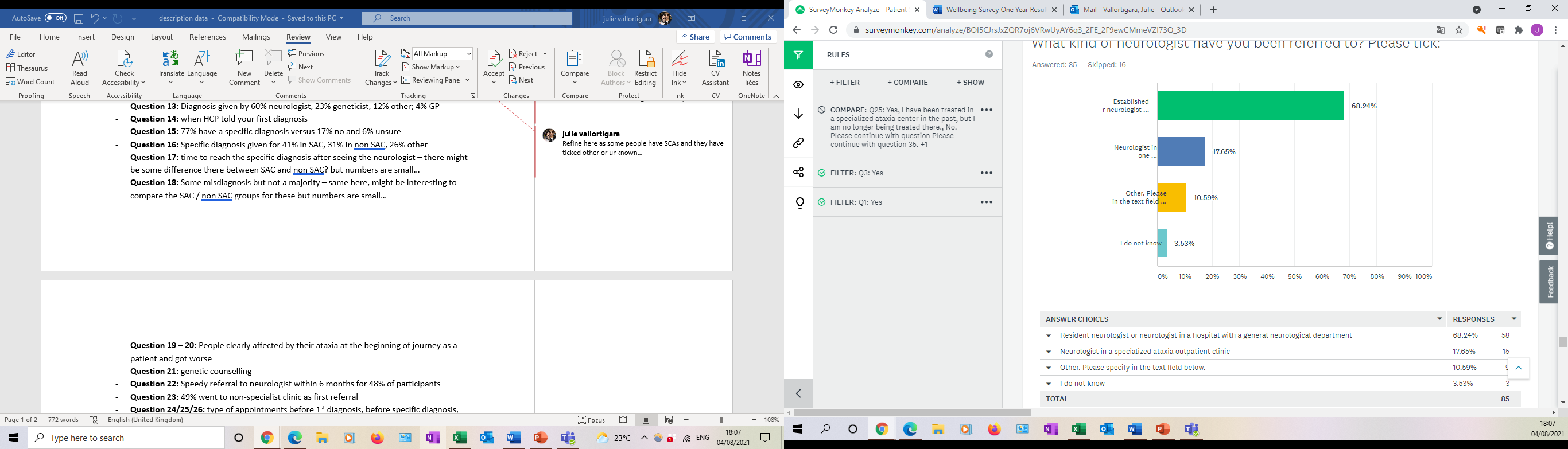


Table 8: participants who went to a non-specialist neurology clinic before going to a SAC


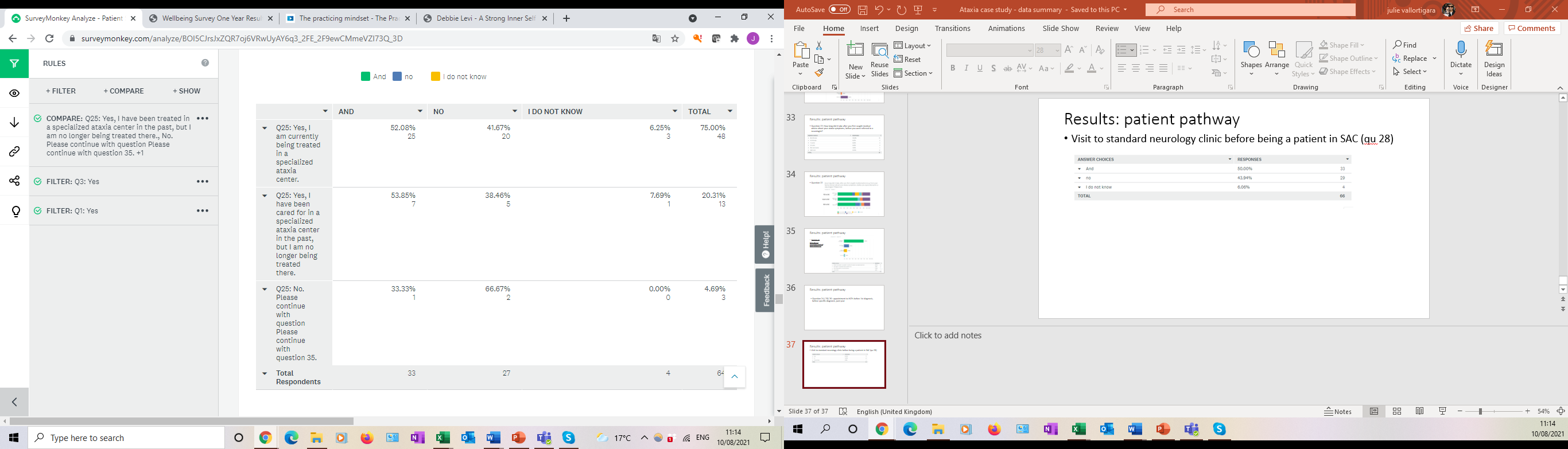


Table 9: Reasons why people stopped going to a SAC


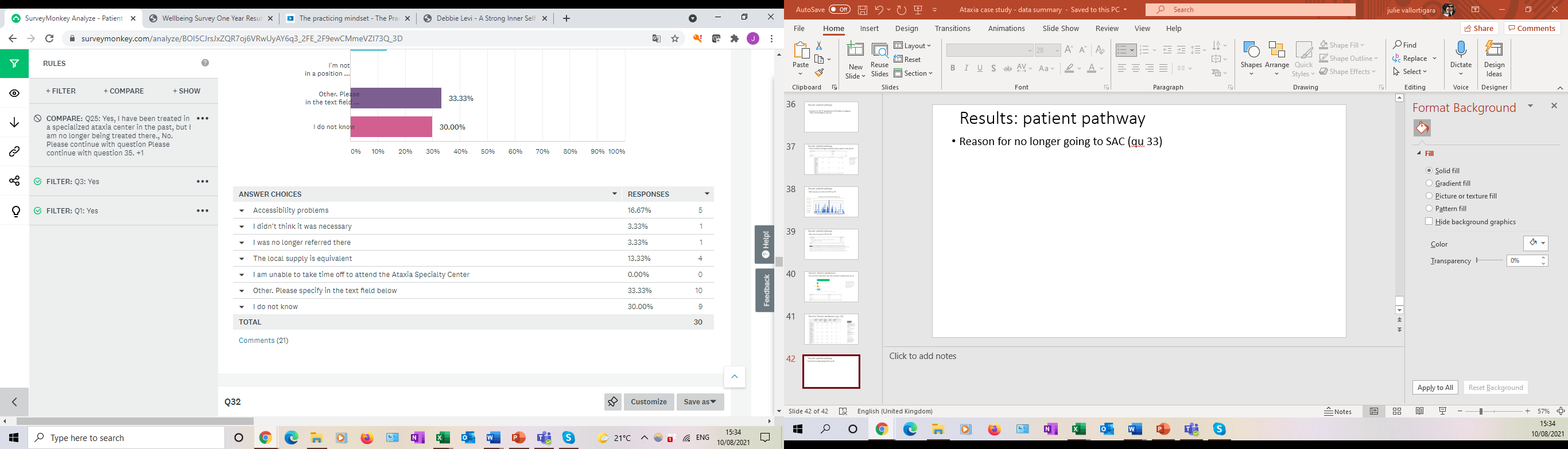


Comments: cancellation due to Covid, diagnosis not verified so only taking part in research, only require to see them every two years, was asked to see a general neurologist, ongoing care (?), attended an appointment at SAC as part of a research study.

Figure 3: Time to travel to a SAC


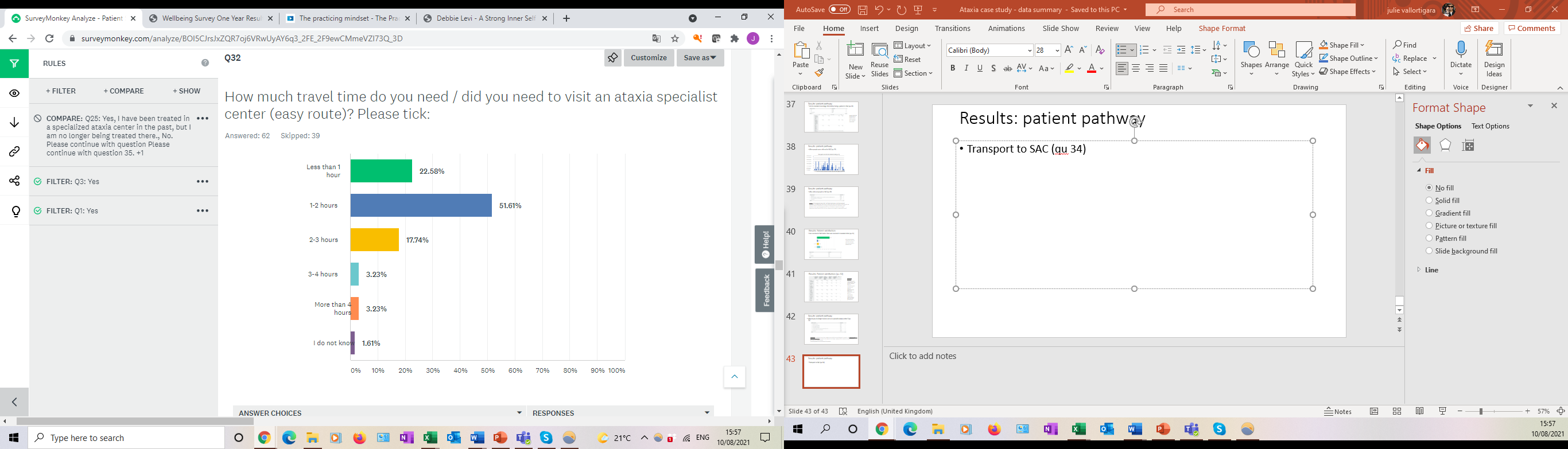


Figure 4: Time to travel to a standard neurology clinic


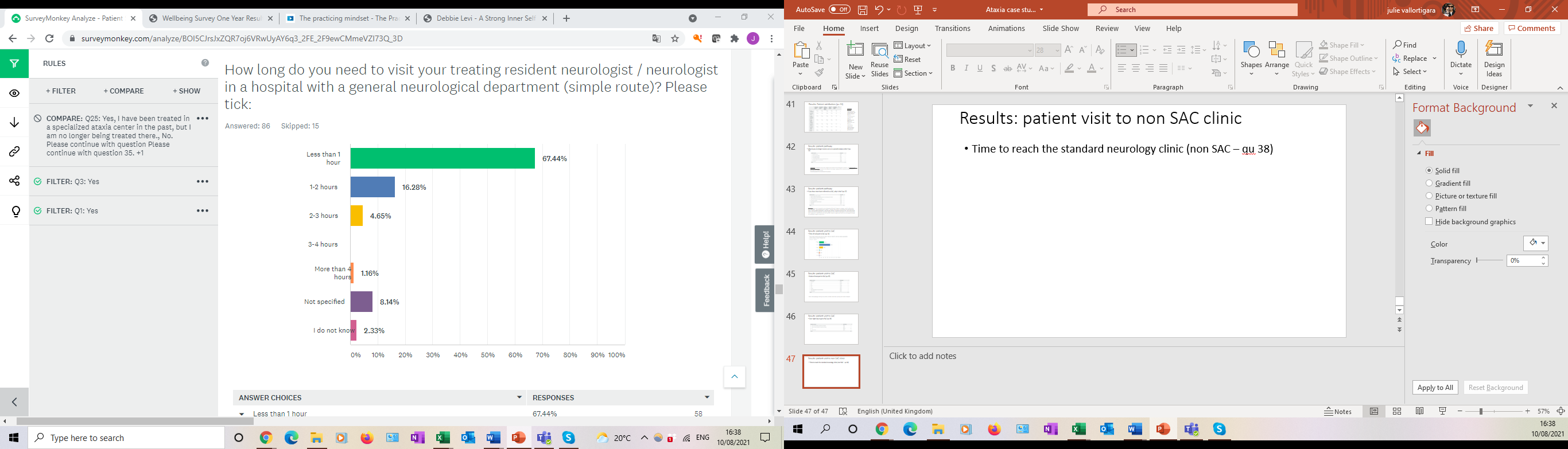


Table 10: Visit to a Multidisciplinary clinic

A: attendance

| **Answer choices** | **Responses N (%)** |
| --- | --- |
| Yes | 12 (16.45%) |
| No | 49 (67.1%) |
| Unsure | 12 (16.45%) |
| **Total** | **73 (100%)** |

B: referral

| **Answer choices** | **Responses N (%)** |
| --- | --- |
| Neurologist at SAC | 1 (9.1%) |
| Neurologist (non-specialist clinic) | 5 (45.45%) |
| Other | 5 (45.45%) |
| **Total** | **11 (100%)** |

Comments: GP referral to physiotherapy, My GP referred me to an inpatient rehabilitation, GP, Feldenkrais physiotherapy, GP, Multidisciplinary Team at rehab clinic, GP,

C: Feedback of participants on the effectiveness of the multidisciplinary team care they received

| Feedback | Positive N (%) | Neutral  N (%) | Negative N (%) |
| --- | --- | --- | --- |
| Number of respondents | 11 (68.75%) | 5 (31.25%) | 0 (0%) |
| Total respondents | 16 (100%) | | |

Table 11: Was the care delivered at a SAC an improvement compared to the care in standard neurology clinic

| Answer choices | responses N (%) |
| --- | --- |
| Yes | 43 (67.2%) |
| No | 4 (6.2%) |
| Non applicable | 6 (9.4%) |
| Unsure | 11 (17.2%) |
| Total | 64 (100%) |

Table 12a: Participants agreement rate on primary care health professionals (e.g. GP, physiotherapist, occupational therapist) understood how to manage their ataxia

| Feedback | Positive N (%) | Neutral  N (%) | Negative N (%) |
| --- | --- | --- | --- |
| YES to SAC | 16 (41%) | 16 (41%) | 7(18%) |
| NO to SAC | 6 (40%) | 7 (47%) | 2 (13%) |
| USED to SAC | 5 (62.5%) | 2 (25%) | 1 (12.5%) |
| Total | 27 (43.5%) | 25 (40.3) | 10 (16.2%) |
| Total respondents | 62 | | |

Table 12b: Participants agreement rate on primary care health professionals (e.g. GP, physiotherapist, occupational therapist) understood the treatments available for their ataxia

| Feedback | Positive N (%) | Neutral  N (%) | Negative N (%) |
| --- | --- | --- | --- |
| YES to SAC | 16 (43.3%) | 13 (35.1%) | 8 (21.6%) |
| NO to SAC | 6 (31.6%) | 8 (42.1%) | 5 (26.3%) |
| USED to SAC | 6 (66.7%) | 3 (33.3%) | 0 (0%) |
| Total | 28 (43%) | 24 (37%) | 13  (20%) |
| Total respondents | 65 | | |

Table 12c: Participants agreement rate on secondary care health professionals (e.g. neurologist, other consultants at my local hospital) understood how to manage their ataxia

| Feedback | Positive N (%) | Neutral  N (%) | Negative N (%) |
| --- | --- | --- | --- |
| YES to SAC | 24 (68.55%) | 8 (22.9%) | 3 (8.55%) |
| NO to SAC | 14 (82.4%) | 3 (14.6%) | 0 (0%) |
| USED to SAC | 8 (100%) | 0 (0%) | 0 (0%) |
| Total | 46 (76.7%) | 11 (18.3%) | 3 (5%) |
| Total respondents | 60 | | |

Table 12d: Participants agreement rate on secondary care health professionals (e.g. neurologist, other consultants at my local hospital) understood the treatments available for their ataxia

| Feedback | Positive N (%) | Neutral  N (%) | Negative N (%) |
| --- | --- | --- | --- |
| YES to SAC | 23 (67.6%) | 9 (26.5) | 2 (5.9%) |
| NO to SAC | 14 (87.5%) | 2 (12.5%) | 0 (0%) |
| USED to SAC | 8 (100%) | 0 (0%) | 0 (0%) |
| Total | 45 (77.6) | 11 (19%) | 2 (3.4%) |
| Total respondents | 58 | | |

Table 12e: Participants agreement rate on the specialists at SAC understood how to manage their ataxia

| Feedback | Positive N (%) | Neutral  N (%) | Negative N (%) |
| --- | --- | --- | --- |
| YES to SAC | 38 (95%) | 2 (5%) | 0 (0%) |
| USED to SAC | 7 (100%) | 0 (0%) | 0 (0%) |
| Total | 45 (95.7%) | 2 (4.3%) | 0 (0%) |
| Total respondents | 47 | | |

Table 12f: Participants agreement rate on specialists at SAC understood the treatments available for their ataxia

| Feedback | Positive N (%) | Neutral  N (%) | Negative N (%) |
| --- | --- | --- | --- |
| YES to SAC | 38 (95%) | 2 (5%) | 0 (0%) |
| USED to SAC | 7 (100%) | 0 (0%) | 0 (0%) |
| Total | 45 (95.7%) | 2 (4.3%) | 0 (0%) |
| Total respondents | 47 | | |

Table 13: How do you feel your care could be improved – by SAC attendance

| **Answer choices** | **SAC**  **N (%)** | **Used to SAC**  **N (%)** | **No to SAC**  **N (%)** | **Total responses** |
| --- | --- | --- | --- | --- |
| More information about my condition | 12 (34.3) | 5 (55.6) | 7 (38/9) | 24 |
| More help so I can feel in control of my disease (to cope better) | 13 (37.1) | 4 (44.4) | 8 (44.4) | 25 |
| Knowing my specific diagnosis earlier | 8 (22.9) | 2 (22.2) | 3 (16.7) | 13 |
| Better management of my symptoms | 12 (34.3) | 4 (44.4) | 4 (22.2) | 20 |
| Better practical advice on living with my condition | 20 (57.1) | 5 (55.6) | 3 (16.7) | 28 |
| Better access to therapies (physiotherapy, speech therapy, occupational therapy) | 11 (31.4) | 2 (22.2) | 4 (22.2) | 17 |
| More information on help adapting my home | 12 (34.3) | 4 (44.4) | 3 (16.7) | 19 |
| Help in communicating with my employer | 6 (17.1) | 0 (0) | 1 (5.6) | 7 |
| More information about the genetics of my condition/ whether my children or grandchildren are at risk of inheriting ataxia | 6 (17.1) | 1 (11.1) | 3 (16.7) | 10 |
| Continuing the same level of care in my home if I am not longer able to visit an ataxia specialist centre | 8 (22.9) | 3 (33.3) | 4 (22.2) | 15 |
| I am satisfied with my care and do not need improvement | 5 (14.3) | 0 (0) | 4 (22.2) | 9 |
| Other please specify | 2 (5.7) | 1 (11.1) | 4 (22.2) | 7 |
| I do not know | 1 (2.9) | 0 (0) | 0 (0) | 1 |
| **Total** | 116 | 31 | 48 | 195 |

**Supplementary Table 14. Health care contacts over a one-year period for non-SAC and SAC patients by number of symptoms experienced as a result of ataxia**

|  | **Patients who reported never attending a SAC** | | | | **Patients who reported attending a SAC currently** | | | |  |  |
| --- | --- | --- | --- | --- | --- | --- | --- | --- | --- | --- |
| **Health care contacts** | **N** | **Mean** | **Std. Dev.** | **Median** | **N** | **Mean** | **Std. Dev.** | **Median** | **P-value**† | **P-value**‡ |
| **No symptoms** |  |  |  |  |  |  |  |  |  |  |
| Specialist centre visits | 1 | 0.0 | - | 0 | 4 | 1.5 | 0.6 | 2 | 0.103 | - |
| Family doctor visits | 1 | 3.0 | - | 3 | 3 | 4.3 | 6.7 | 1 | 0.878 | - |
| Neurologist visits | 1 | 1.0 | - | 1 | 2 | 3.0 | 1.4 | 3 | 0.454 | - |
| Inpatient stays | 2 | 0.0 | 0.0 | 0 | 2 | 0.5 | 0.7 | 1 | 0.423 | - |
| Emergency room visits | 1 | 1.0 | - | 1 | 2 | 0.0 | 0.0 | 0 | - | - |
| Physiotherapy visits | 2 | 25.0 | 35.4 | 25 | 1 | 50.0 |  | 50 | 0.667 | - |
| Speech and language therapy visits | 1 | 0.0 | - | 0 | 5 | 10.2 | 17.2 | 1 | 0.617 | 0.788 |
| Occupational health therapy visits | 1 | 0.0 | - | 0 | 2 | 5.0 | 7.1 | 5 | 0.667 | - |
| Other consultant specialist visits | 1 | 5.0 | - | 5 | 3 | 2.0 | 1.7 | 1 | 0.272 | - |
| Total cost | 1 | 621 | - | 621 | 1 | 3155 | - | 3155 | - | - |
| **1-4 symptoms** |  |  |  |  |  |  |  |  |  |  |
| Specialist centre visits | 6 | 0.0 | 0.0 | 0 | 18 | 1.1 | 0.2 | 1 | <0.01 | <0.01 |
| Family doctor visits | 3 | 10.3 | 17.0 | 1 | 19 | 4.0 | 4.1 | 3 | 0.14 | 0.217 |
| Neurologist visits | 6 | 4.3 | 4.0 | 3 | 18 | 2.3 | 3.2 | 2 | 0.224 | 0.251 |
| Inpatient stays | 5 | 0.0 | 0.0 | 0 | 19 | 0.3 | 0.8 | 0 | 0.406 | 0.32 |
| Emergency room visits | 5 | 0.0 | 0.0 | 0 | 18 | 0.2 | 0.6 | 0 | 0.458 | 0.381 |
| Physiotherapy visits | 6 | 36.8 | 20.3 | 48 | 17 | 31.8 | 24.2 | 50 | 0.656 | 0.884 |
| Speech and language therapy visits | 6 | 6.5 | 15.0 | 0 | 17 | 12.4 | 18.4 | 3 | 0.488 | 0.038 |
| Occupational health therapy visits | 6 | 13.2 | 21.3 | 0 | 17 | 10.1 | 18.0 | 0 | 0.736 | 0.594 |
| Other consultant specialist visits | 5 | 1.6 | 2.5 | 1 | 15 | 1.8 | 2.5 | 1 | 0.88 | 0.715 |
| Total cost | 3 | 2448 | 1983 | 3560 | 14 | 4652 | 4758 | 2844 | 0.452 | 0.263 |
| **5-8 symptoms** |  |  |  |  |  |  |  |  |  |  |
| Specialist centre visits | 0 | - | - | - | 5 | 2.0 | 2.2 | 1 | - | - |
| Family doctor visits | 0 | - | - | - | 5 | 10.8 | 7.3 | 12 | - | - |
| Neurologist visits | 0 | - | - | - | 5 | 4.0 | 3.2 | 4 | - | - |
| Inpatient stays | 0 | - | - | - | 4 | 0.3 | 0.5 | 0 | - | - |
| Emergency room visits | 0 | - | - | - | 5 | 3.0 | 6.7 | 0 | - | - |
| Physiotherapy visits | 0 | - | - | - | 4 | 22.8 | 16.6 | 25 | - | - |
| Speech and language therapy visits | 0 | - | - | - | 5 | 8.4 | 17.7 | 0 | - | - |
| Occupational health therapy visits | 0 | - | - | - | 5 | 10.4 | 22.2 | 0 | - | - |
| Other consultant specialist visits | 0 | - | - | - | 4 | 1.5 | 1.0 | 2 | - | - |
| Total cost | 0 | - | - | - | 3 | 5193 | 3128 | 3785 | - | - |
| **P-value**§ |  |  |  |  |  |  |  |  |  |  |
| Family doctor visits | 0.212 |  |  |  | 0.079 |  |  |  |  |  |
| Neurologist visits | 0.281 |  |  |  | 0.493 |  |  |  |  |  |
| Inpatient stays | - |  |  |  | 0.578 |  |  |  |  |  |
| Emergency room visits | - |  |  |  | 0.109 |  |  |  |  |  |
| Physiotherapy visits | 0.757 |  |  |  | 0.800 |  |  |  |  |  |
| Speech and language therapy visits | - |  |  |  | 0.885 |  |  |  |  |  |
| Occupational health therapy visits | - |  |  |  | 0.427 |  |  |  |  |  |
| Other consultant specialist visits | 0.286 |  |  |  | 0.634 |  |  |  |  |  |
| Total cost | 0.746 |  |  |  | 0.849 |  |  |  |  |  |

† Test for significant differences in mean values between Non-SAC and SAC groups (unadjusted)

‡ Test for significant differences in mean values between Non-SAC and SAC groups (adjusted for age, sex and comorbidities)

§ Test for significant differences in mean values by number of symptoms separately for Non-SAC and SAC groups (adjusted for age, sex and comorbidities)

A hyphen “-“ indicates that the parameter is not estimable, due to small numbers of observations.

SAC, specialist ataxia centre; N, number of participants who responded to that question.

**Table 15. Mode of transport and time taken to travel to the SAC and the neurologist based in the general neurology clinic**

| **Travel time to visit the Specialist Ataxia Centre (one way)** | | | | **Mode of transport mainly used to visit the SAC** | | | | |
| --- | --- | --- | --- | --- | --- | --- | --- | --- |
|  | **N** | **%** | |  | | **N** | | **%** |
| Less than 1 hour | 10 | 22 | | Bus | | 4 | | 9 |
| 1 to 2 hours | 26 | 57 | | Car | | 32 | | 68 |
| 2 to 3 hours | 7 | 15 | | NHS transport | | 2 | | 4 |
| 3 to 4 hours | 2 | 4 | | Taxi | | 3 | | 6 |
| More than 4 hours | 1 | 2 | | Train | | 6 | | 13 |
| **Travel time to visit the neurologist based in the general neurology clinic (one way)** | | | | | | | | |
|  | **Patients who reported never attending a SAC** | | | | **Patients who reported attending a SAC currently** | | | |
|  | **N** | | **%** | | **N** | | **%** | |
| Less than 1 hour | 18 | | 86 | | 30 | | 63 | |
| 1 to 2 hours | 2 | | 10 | | 7 | | 15 | |
| 2 to 3 hours | 1 | | 4 | | 3 | | 6 | |
| Not applicable | 0 | | 0 | | 6 | | 13 | |
| Unsure | 0 | | 0 | | 1 | | 2 | |
